# Dynamics of *Verticillium dahliae* race 1 population under managed agricultural ecosystems

**DOI:** 10.1186/s12915-021-01061-w

**Published:** 2021-06-25

**Authors:** Jie-Yin Chen, Dan-Dan Zhang, Jin-Qun Huang, Ran Li, Dan Wang, Jian Song, Krishna D. Puri, Lin Yang, Zhi-Qiang Kong, Bang-Zhuo Tong, Jun-Jiao Li, Yu-Shan Huang, Ivan Simko, Steven J. Klosterman, Xiao-Feng Dai, Krishna V. Subbarao

**Affiliations:** 1 State Key Laboratory for Biology of Plant Diseases and Insect Pests, Institute of Plant Protection, Chinese Academy of Agricultural Sciences, Beijing, China; 2 BGI-Shenzhen, Shenzhen, Guangdong China; 3 Department of Plant Pathology, University of California, Davis, c/o U.S. Agricultural Research Station, Salinas, CA USA; 4 United States Department of Agriculture, Agricultural Research Service, Crop Improvement and Protection Research Unit, Salinas, CA USA

**Keywords:** *Verticillium dahliae*, Managed agricultural ecosystems, Local adaptation, Genetic selection, Transposon enrichment, Signal transduction, Transcriptional regulation

## Abstract

**Background:**

Plant pathogens and their hosts undergo adaptive changes in managed agricultural ecosystems, by overcoming host resistance, but the underlying genetic adaptations are difficult to determine in natural settings. *Verticillium dahliae* is a fungal pathogen that causes Verticillium wilt on many economically important crops including lettuce. We assessed the dynamics of changes in the *V. dahliae* genome under selection in a long-term field experiment.

**Results:**

In this study, a field was fumigated before the *Verticillium dahliae* race 1 strain (VdLs.16) was introduced. A derivative 145-strain population was collected over a 6-year period from this field in which a seggregating population of lettuce derived from *Vr1/vr1* parents were evaluated. We de novo sequenced the parental genome of VdLs.16 strain and resequenced the derivative strains to analyze the genetic variations that accumulate over time in the field cropped with lettuce. Population genomics analyses identified 2769 single-nucleotide polymorphisms (SNPs) and 750 insertion/deletions (In-Dels) in the 145 isolates compared with the parental genome. Sequence divergence was identified in the coding sequence regions of 378 genes and in the putative promoter regions of 604 genes. Five-hundred and nine SNPs/In-Dels were identified as fixed. The SNPs and In-Dels were significantly enriched in the transposon-rich, gene-sparse regions, and in those genes with functional roles in signaling and transcriptional regulation.

**Conclusions:**

Under the managed ecosystem continuously cropped to lettuce, the local adaptation of *V. dahliae* evolves at a whole genome scale to accumulate SNPs/In-Dels nonrandomly in hypervariable regions that encode components of signal transduction and transcriptional regulation.

**Supplementary Information:**

The online version contains supplementary material available at 10.1186/s12915-021-01061-w.

## Background

Evolution of pathogens occurs by local adaptation in both natural and agricultural ecosystems [1]. In the process, pathogens are thought to evolve more rapidly than their hosts owing to their larger population sizes, shorter generation times, and more plastic mutation mechanisms. As a consequence, pathogen populations may adapt more quickly than their host counterparts in a coevolutionary arms race [2, 3]. The mechanisms of local adaptation differs between natural and agricultural ecosystems. In the natural ecosystems, the selection pressure is tempered by host and environmental heterogeneity as well as pathogen trade-offs between pathogenicity and lifestyle traits, which results in the selection of genetic variation at the genomic and population levels in both plants and pathogens [4, 5]. However, pathogens may confront greater challenges in the agricultural ecosystems even though they appear to provide a homogeneous environment owing to selection pressures imposed on pathogen populations, including the variable conditions of fertilization, irrigation, pesticide applications, tillage, and soil physical and chemical properties [3].

A diversity of factors in agricultural ecosystems also play important roles in local adaptation [3]. For instance, quinone outside inhibiting (QoI) fungicides represent one of the most widely used groups of fungicides to control agriculturally important fungal pathogens by targeting the respiratory chain, but the mutation in cytochrome-b led to the emergence of QoI fungicide-resistant isolates of *Plasmopara viticola* [6]. Similarly, temperature has been shown to be a key abiotic factor leading to local adaptation in agricultural ecosystems [7]. For instance, *Zymoseptoria tritici* populations collected from warm environments showed faster growth than the populations collected from cold environments [3, 8]. Therefore, local adaptation of host plants and pathogens is facilitated by conditions that are heavily manipulated in agricultural ecosystems, resulting in the evolution of pathogens highly successful in different geographic regions [3].

Adaptations of pathogens in the agricultural ecosystem may also include those involving effector genes which are under strong positive selection that may be highly detrimental or beneficial for the pathogen [9]. To a large extent, this selection determines the success or failure of host resistance in agricultural ecosystems. The coevolutionary arms race drives adaptation of pathogen effectors and host resistance genes, supported by the gene-for-gene (avirulence gene *vs* resistance gene) theory [10]. For instance, the introgression of resistance genes *Rlm1* and *Stb4* into *Brassica napus* cultivars resulted in *Leptosphaeria maculans* avirulence genes evolving in agricultural systems. However, in most cases, the local adaptation of pathogens is largely determined by genes other than a member of the gene-for-gene interactions governing plant pathogen-host interactions. Examples include *Rhynchosporium commune* [1], *Zymoseptoria tritici* [11], and *Parastagonospora nodorum* [12]. The arms race evolution drives the continuous replacement of alleles (selective sweep) in the pathogen population in response to the continuous emergence of new resistance alleles in the host under the agricultural ecosystems [13]. For instance, the encoded proteins of strongest selective sweep regions in *Rhynchosporium commune*, regulate functions related to biotic and abiotic stress responses, in contrast to the prevailing view that a small number of gene-for-gene interactions governing plant pathogen evolution [1]. In addition, the selection for local adaptation is generally mediated by the plasticity in the genome [5]. As an example, transposable elements (TE) with high mutagenic potential lead to chromosomal variations and drive changes to both the protein repertoire and regulatory networks in prokaryotic and eukaryotic genomes in the agricultural ecosystems [14]. TEs serve as an active site of evolution in *Aspergillus niger* [15], *Cochliobolus carbonum* [16], and *Magnaporthe oryzae* [17]. Such elements undergo selective amplification following integration into the genome, and some are acquired through horizontal gene transfer [18–21]. TE-mediated gene losses may facilitate host range expansion as in *M. oryzae* [22] or loss of host-specific virulence as in *Alternaria alternata* [23].

*Verticillium dahliae* is an asexually reproducing, soilborne fungus that causes vascular wilt in over 200 plant species, including many economically important crops [24–26]. The fungus forms melanized resting structures called microsclerotia, which remain viable in the soil in the absence of hosts for up to 14 years and serve as primary inoculum for subsequent crops [27, 28]. The microsclerotia germinate in response to signals from host root exudates and initiate infection through roots [28–30]. Even when challenged with host resistance, *V. dahliae* is capable of infecting epidermal cells for limited proliferation [31, 32]. *Verticillium dahliae* produces an exoproteome that plays important roles during colonization and proliferation in the vascular system, disease establishment, and symptom development [27, 33, 34].

Lettuce *Vr1* is the resistance locus against the avirulence gene *Ave1* of race 1 in *V. dahliae* [35]. In a previous study, seven simple sequence repeat (SSR) variants were identified among 427 members of a clonal population of VdLs.16 (race 1) collected over 6 years from a field infested with the race 1 VdLs.16 strain and planted with a segregating population of lettuce derived from *Vr1/vr1* parents*.* The results offered no evidence for altered mating-type, virulence loci, or phenotypes in this managed agricultural ecosystem [36]. However, the study was limited to the evaluation of variation in the seven SSR loci distributed on five out of eight chromosomes. Despite the higher mutation rate, multiallelic nature, and high variability of SSR markers, they are unlikely to fully capture the genome-wide changes [37–39] in this managed agricultural ecosystem. Therefore, the genetic basis of local adaptation, including the environment and host selection in *V. dahliae* in fields continuously planted with resistant lettuce within a managed agricultural ecosystem, is unknown.

The overall aim of this study was to investigate the occurrence of genome-wide variations in a field population of *V. dahliae* VdLs.16 strain and 145 isolates derived from this strain that were collected over 6 years in a lettuce disease nursery [36]. To accomplish this aim, the whole genomes of the 145 isolates were resequenced. The specific objectives of this study were to (1) perform comparative genomic analysis of VdLs.16 genome with the reference genomes of JR2 (race 1 from tomato) and VdLs17 (race 2 from lettuce); (2) examine the potential evolution on the single clonal population of *V. dahliae* exploring genome-wide single-nucleotide polymorphisms (SNPs), insertions-deletions (In-Dels), and other trackable genetic variations; and (3) to identify genes under selective pressure and ongoing genetic fixation in *V. dahliae* in a managed agricultural ecosystem.

## Results

### Sequenced genome of the VdLs.16 strain

To study the local adaptation of VdLs.16 strain under the managed agricultural ecosystem, the whole genome of VdLs.16 strain was assembled de novo and initially compared with the reference genomes of JR2 (race 1) and VdLs.17 (undetermined race, since it lacks both *Ave1* and *Av2* that characterize race 1 and 2, respectively) [40, 41]. The genome of VdLs.16 strain, previously characterized as race 1 from lettuce [42], was assembled from sequence data derived from PacBio RS II and Illumina sequencing technologies (Additional file 2: Table S1). The genome size of VdLs.16 strain assembled was 36.09 Mb and was composed of 14 scaffolds with the N50 of 4.72 Mb and a GC content of 53.8% (Table 1). By combining automated gene calling from de novo-based and homology-based prediction methods, the VdLs.16 genome was predicted to encode 10,799 genes, very similar to the total re-predicted genes in JR2 and VdLs.17 genomes (Table 1). More than 10% of the sequence (10.7% of VdLs.16 genome) was composed of TEs among the three *V. dahliae* genomes. Nearly 30% of the TEs (1.15 Mb) were of the LTR/Gypsy type (Additional file 2: Table S2). The functional annotation of protein-coding genes in the VdLs.16 genome revealed 797 secreted proteins, 588 carbohydrate-active enzymes (CAZymes) of which 266 were predicted as secreted (Additional file 2: Tables S3–S6), and an arsenal of pathogenicity and virulence-related genes similar to those in the JR2 and VdLs.17 genomes (Table 1). Among the secreted proteins, 302 encoded small cysteine-rich proteins (SCRPs) were detected. The annotation also revealed 150 protein kinases (PKs) (Additional file 2: Table S7) and 2797 encoded homologs of pathogen-host interaction (PHI) proteins (Additional file 2: Table S8). The numbers of predicted transcription factors (TF) were significantly different among the three *V. dahliae* genomes, which encode 432, 482, and 500 TFs in VdLs.16, JR2, and VdLs.17 genomes, respectively (Table 1; Additional file 2: Table S9). Despite these differences, the overall genome size and the content of VdLs.16 was highly similar to the previously released genomes of isolates from lettuce, tomato, and cotton [21, 43, 44].

**Table 1:** Genome assembly statistics for three strains of *Verticillium dahliae*

Strain		VdLs.16		JR2		VdLs.17	
Genome assembly		Scaffold	Contig	Scaffold	Contig	Scaffold	Contig
	Total number	14	17	8	8	8	8
	Total length of (bp)	36,092,517	36,089,483	36,150,287	36,150,287	35,973,870	35,973,870
	Gap number (bp)	3034	–	0	–	0	–
	Average length (bp)	2,578,037	2,122,911	4,518,786	4,518,786	4,496,734	4,496,734
	N50 length (bp)	4,722,744	3,663,785	4,168,633	4,168,633	5,894,008	5,894,008
	N90 length (bp)	2,532,149	2,375,583	3,361,230	3,361,230	3,290,897	3,290,897
	Maximum length (bp)	8,087,087	8,087,087	9,275,483	9,275,483	6,210,300	6,210,300
	Minimum length (bp)	50,807	41,068	3,277,570	3,277,570	3,272,870	3,272,870
	GC content (%)	53.8	53.8	53.88	53.88	54	54
Genome prediction	Protein-coding genes	10,799		10,875		10,825	
	Length of genes (bp)	18,863,221		19,050,607		19,146,372	
	Mean gene length (bp)	1,747		1752		1769	
	Gene GC %	58.56		58.49		58.46	
	Length of CDS (bp)	16,527,821		16,720,002		16,761,722	
	Mean length of CDS (bp)	1530		1537		1548	
	CDS GC %	59.41		59.31		59.29	
	Exon no.	31,232		31,303		31,696	
	Coding gene with introns	1.89		1.87		1.92	
	Mean exons per gene	2.89		2.87		2.92	
	Mean exons length (bp)	529		534		529	
	Intron no.	20,433		20,428		20,871	
	Mean intron length (bp)	114		114		114	
	Intron GC %	52.26		52.25		52.29	
	Mean intergenic length (bp)	1584		1571		1553	
	No. of transposons	4870		4665		5262	
	Total length of transposons (bp)	3,879,742		4,057,098		4,803,307	
	Percentage in genome	10.7494		11.2228		13.3522	
Genome annotation	Secretome	797		803		803	
	CAZymes	588		589		590	
	Pectin degradation	36		35		35	
	Cellulose degradation	84		84		82	
	Hemicellulose degradation	67		67		65	
	Ligin degradation	4		5		4	
	CAZymes (secreted)	266		268		270	
	SCRPs	302		313		297	
	LysM effectors	6		6		7	
	NLPs	7		7		8	
	Protein kinases	150		156		154	
	Transcription factors	432		482		500	
	PHI homologs	2797		2778		2789	

### Whole-genome resequencing of the VdLs.16 population under selection in managed agricultural ecosystems

To identify the genetic changes that occur in response to local adaptation in *V. dahliae*, a derivative VdLs.16 population was collected from symptomatic roots samples from the field that evaluated a resistant (carrying resistance gene *Vr1* against *Ave1* of race 1 strain) × susceptible (*vr1* genotype) segregating population of lettuce during 2010–2015 [45], and 145 strains were selected for genome resequencing in this study (Additional file 2: Table S10). The genomes of these strains were resequenced by the Illumina platform, generating more than 3 Gb of 150 bp paired-end reads (Additional file 2: Table S11). Read mapping showed that > 99% of the parental VdLs.16 genome was covered to a depth of ≥ 20× for each strain (Additional file 2: Table S12). The coverage and depth of reads mapped to the parental genome revealed that the sequenced derivative strains and the parental race 1 strain contained the characteristic markers for race 1 (*Ave1*), non-defoliating phenotype, and mating-type 2 (*MAT1-2*) (Additional file 2: Table S13). The derived VdLs.16 isolates therefore were a representative population to analyze the evolution of *V. dahliae* in a managed agricultural ecosystem.

### Genetic variations in the VdLs.16-derived population relative to the parental strain

**Fig. 1:**
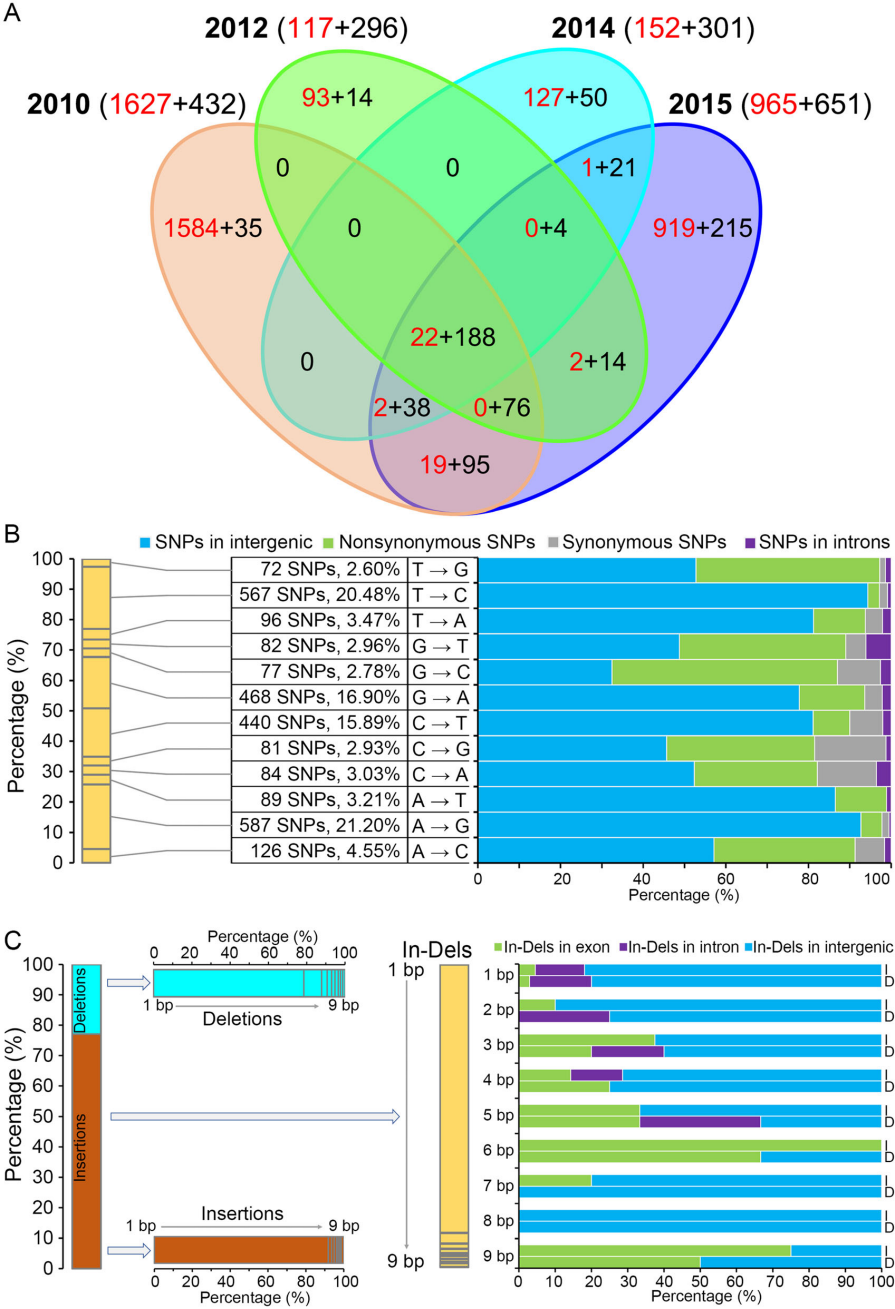
Accumulation of the genetic variations in a *Verticillium dahliae* population in a managed agricultural ecosystem. The genome of the parental race 1 strain of *V. dahliae* (VdLs.16) was set as the reference to call the genetic variations of single-nucleotide polymorphisms (SNPs) and insertions and deletions (In-Dels). The variations were called from the VdLs.16 population that was collected from a field planted with race 1-resistant lettuce over 6 years. **a** Statistics of the genetic variations from the VdLs.16 population collected in 2010, 2012, 2014, and 2015. The numbers in red and black letters represent the total amount of SNPs and In-Dels in VdLs.16 population, respectively. **b** Identification of SNPs in the VdLs.16 population. **c** Collection of the insertions and deletions (In-Dels) in the VdLs.16 population. The letters “I” and “D” represent the genetic variations of insertions and deletions, respectively

To detect the sequence divergence among the isolates derived from VdLs.16, single-nucleotide polymorphisms (SNPs) and insertions-deletions (In-Dels) were compared by mapping the high coverage and depth of sequenced reads to the VdLs.16 reference genome. In total, only 3519 genetic variations were called from the VdLs.16 population, including 2769 SNPs with four main mutation types (A → G, T → C, G → A, and C → T) and 750 In-Dels (1 – 9 bp, predominantly 1 bp) (Fig. 1a; Additional file 2: Tables S14-S16). Most SNP variations were detected in VdLs.16-derived population collected in 2010, which possessed 58.75% (1627) SNPs (Fig. 1a). Only 0.7% (22 SNPs ) were common among the collected strains across 4 years, 97.4% (1584 SNPs) in 2010, 79.5% (93 SNPs) in 2012, 83.6% (127 SNPs) in 2014, and 95.2% (919 SNPs) in 2015 were specific to individual years (Fig. 1a). However, In-Del variation displayed higher consolidation than for SNPs with 25.1% (188) of In-Dels commonly detected over four sampling years (Fig. 1a). The SNP variations of adenine (A) substituted for guanine (G) were 21.2% and thymine (T) substituted for cytosine (C) were 20.48% of the total substitutions detected in the derived population (Fig. 1b). The 1 bp In-Del was the most predominant in the VdLs.16-derived population (Fig. 1c). In addition, analyses of genetic variations among the various loci showed that 13.94% (386) of nonsynonymous SNPs and 6.93% (52) of In-Dels were located on the exons (Additional file 1: Figure S1A), whereas most of the genetic variations occurred in the intergenic regions (Fig. 1b, c; Additional file 2: Tables S14 and S15).

### Divergence caused by the genetic variations in the VdLs.16 population

**Fig. 2:**
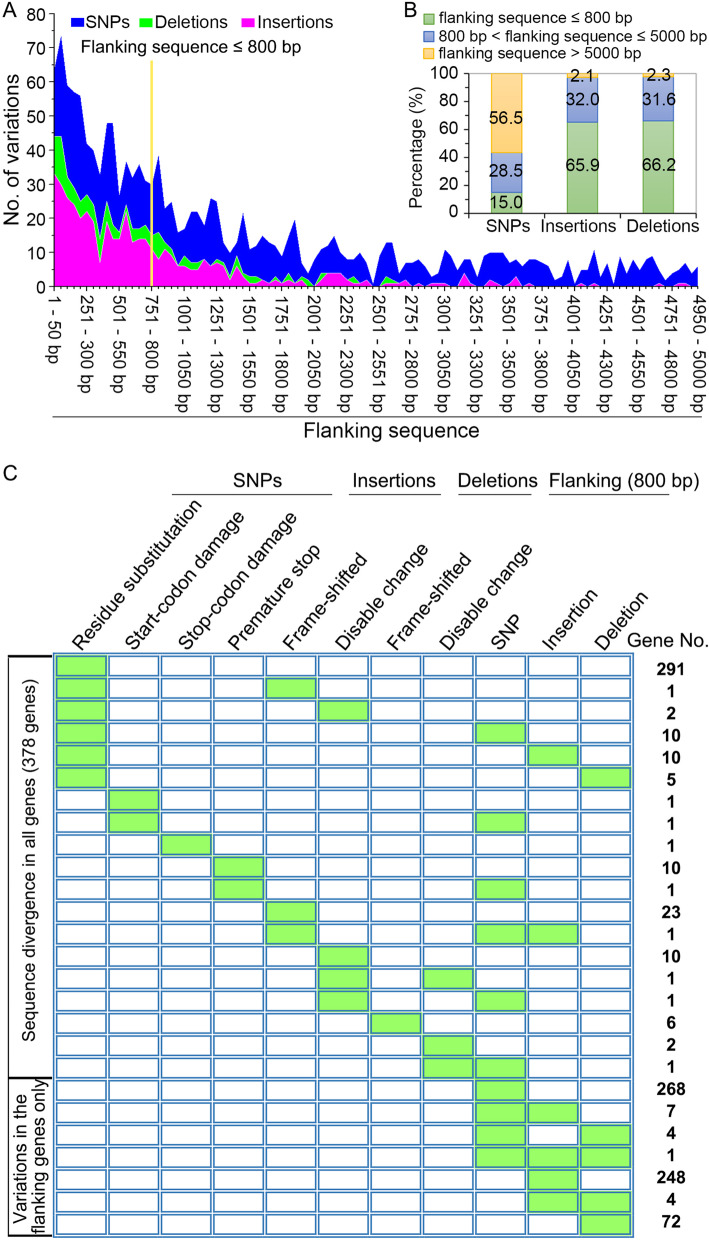
Genes under selection for local adaptation in the genome of strain VdLs.16 of *Verticillium dahliae*. **a** Distribution of the intergenic SNPs and In-Dels in the gene flanking sequences of 5000 bp. The yellow line is the boundary line separating the 5′ upstream 800 bp flanking sequence from the remainder of the upstream flanking sequence. **b** Percentage of the intergenic genetic variations among the gene flanking sequences. **c** Genes under selection determined by the analysis of genetic variation in the VdLs.16 genome. The green colored blocks represent the gene divergence caused by particular genetic changes. The 5′ upstream 800 bp with genetic variation was also defined as under selection. These mutations are nonsense, multiples of 3 bp deletions or insertion, but no frame shifts

The genetic variation included both coding sequence changes (nonsynonymous SNPs and In-Dels in exons) and changes in the predicted gene promoter regions (800 bp of 5′ sequence) in response to local adaptation. There were 386 nonsynonymous SNPs and 52 In-Dels in the coding sequences (Additional file 1: Figure S1A). The location of the genetic variations in the intergenic region revealed that 725 (20.6%) variations, including 332 SNPs (11.99% of total SNPs) and 393 In-Dels (52.4% of total In-Dels) (Additional file 1: Figure S1A; Additional file 2: Table S16), were located in the predicted promoter sequences within 800 bp upstream of the open reading frames of the predicted genes (Fig. 2a). In-Dels in the intergenic region included 65.9% of insertions and 66.2% of deletions, while 15% of SNPs in the intergenic regions were located within the 800 bp upstream flanking sequence (Fig. 2b), suggesting that the In-Dels were more commonly associated with the predicted promoter sequences.

There were 982 predicted genes in the derivative population that had diverged from the parental VdLs.16 isolate, based on the nonsynonymous SNPs and In-Dels in exon and gene promoter sequences (Fig. 2a; Additional file 2: Table S17). Among the 378 (38.49%) predicted genes with sequence divergence (mutation) in their exons, 30 also contained mutations in their predicted promoter sequences (Fig. 2b; Additional file 1: Figure S1B). Within the mutated gene set, the sequence divergence was primarily characterized by nonsynonymous SNPs (76.99%, 291 genes) in exons (Fig. 2b). Moreover, 604 genes (61.51%) lacked mutations in their coding sequences but contained variations in their predicted promoter sequences. Among these, there were 268 SNPs and 248 insertions (Fig. 2b). Analyses of the *Ave1* locus showed no variations in the derived population (Additional file 1: Figure S2), even though the VdLs.16 strain was under host selection. These results indicated that the VdLs.16 genome underwent field/host-induced changes, defined in part by sequence divergence within the predicted encoded genes or the predicted promoter sequences.

### Functional analysis of genes under selection pressure in the VdLs.16 population

**Fig. 3:**
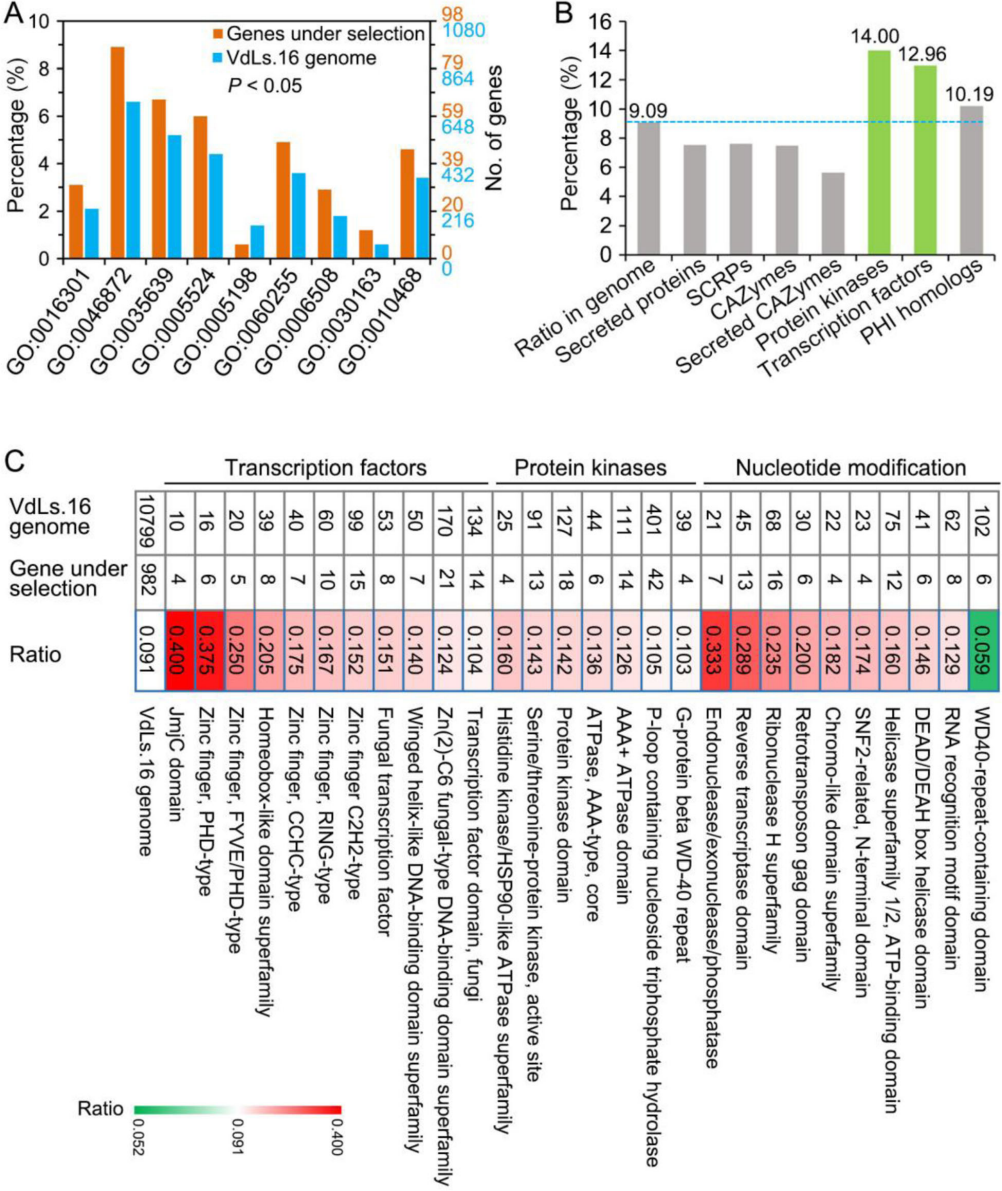
Functional annotation of genes under selection for local adaptation in the genome of *Verticillium dahliae* strain VdLs.16. **a** Gene ontology (GO) annotation of the genes under selection in VdLs.16 genome. Significant GO catalogs of genes under selection pressure in the VdLs.16 population were compared to the whole genome were determined by the Pearson chi-square test (*P* < 0.05). GO:0016301, kinase activity; GO:0046872, metal ion binding; GO:0035639, purine ribonucleoside triphosphate binding; GO:0005524, ATP binding; GO:0005198, structural molecule activity; GO:0060255, regulation of macromolecule metabolic process; GO:0006508, proteolysis; GO:0030163, protein catabolic process; GO:0010468, regulation of gene expression. **b** Functional annotation of the genes in VdLs.16 genome under selection with potential roles in pathogenicity. The column designated as “ratio in genome” represents the ratio of genes under selection relative to the total number of encoded genes in the VdLs.16 genome; the other columns represent the percentage of certain annotation type compared to the genes of that type under selection. **c** Annotation of the genes under selection in the VdLs.16 population

The genes under local adaptation included those significantly enriched in regulatory function, including regulation of gene expression (GO:0030163, 45 genes), kinase activity (GO:0016301, 30 genes), regulation of macromolecule metabolic processes (GO:0060255, 48 genes) (*P* < 0.05) (Fig. 3a), suggesting that the gene functions associated with regulatory roles responded to local adaptation relative to other genes in the VdLs.16 field population. Annotation of the genes in the VdLs.16 field population with potential pathogenicity functions revealed that 60 of these encoded secreted proteins including 23 SCRPs and 15 secreted CAZymes (Fig. 3b). In addition, 21, 56, and 251 genes sharing homology with PKs, TFs, and those encoding 285 PHI related proteins (Fig. 3b), respectively, were also impacted. The genes encoding PKs and TFs, as a ratio of mutated versus total annotated (14.0% and 12.96% of the PKs and TFs, respectively), were also higher than the whole genome (9.09%) (Fig. 3b), suggesting that the genes governing regulatory processes may undergo changes more frequently. The TF family of zinc finger (PHD-type, CCHC-type, etc.) and the PK families of histidine kinase and serine/threonine-protein kinases were under significantly higher selection pressure than others (ratio of gene number under selection in each family was higher than the average in the parental genome) in the VdLs.16 genome (Fig. 3c). Several gene families involved in nucleic acid modification also displayed higher overall mutational rates than the parental genome. These included endonuclease, reverse transcriptase, and ribonuclease (Fig. 3c) potentially involved in nucleotide repair during sequence divergence/mutation in the VdLs.16 strain under selection in managed agricultural ecosystems.

### Genetic variations are significantly enriched in the transposon-rich and gene-sparse regions

**Fig. 4:**
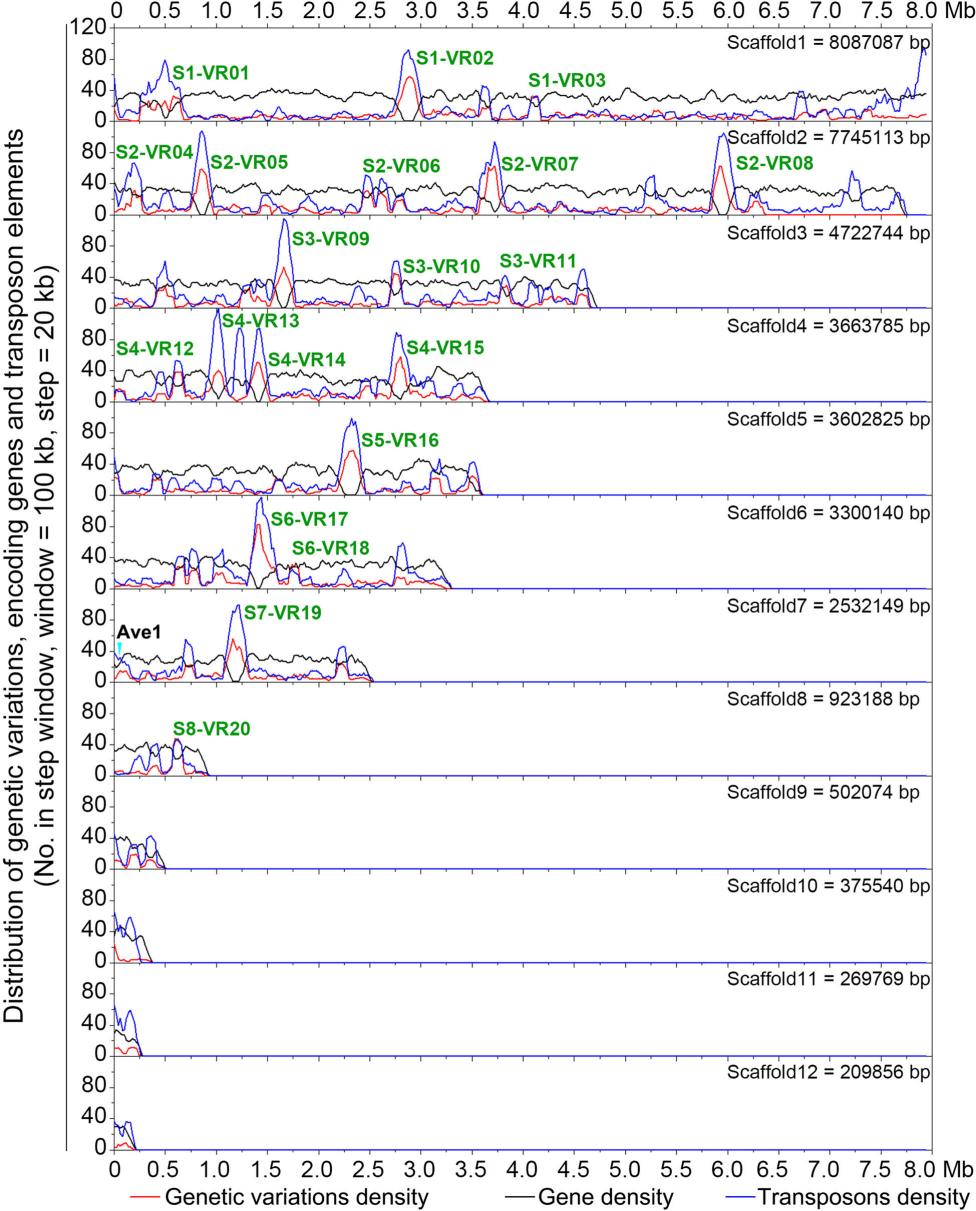
Regions of increased genetic variations (RIVs) and associated genes and transposons in the genome of strain VdLs.16 of *Verticillium dahliae*. Curves were drawn according to the density of genetic variation, in which encoded genes and transposable elements were presented in windows of in 100 kb with 20-kb steps. The window contains up to 30 genetic variations (including SNPs and In-Dels) defined as the region of genetic variation

To identify the locations in the genome that undergo variations in the derivative population, the distribution of the SNPs and In-Dels was investigated by mapping them onto the VdLs.16 genome in stepwise incremental windows (window = 100 kb, step = 20 kb). The distribution of genetic variation present in the VdLs.16 genome was dispersed into 20 regions of increased variation (RIVs) in windows containing more than 30 SNPs and In-Dels (Fig. 4; Additional file 2: Table S18). The stepwise windows analysis revealed that fewer genes and higher numbers of TEs among RIVs relative to the core genome (Fig. 4). While the RIVs comprised only 12.4% of the VdLs.16 genome length (4.46 Mb) and contained fewer genes (8%, 861 genes in RIVs), they accounted for 36.9% of the SNPs and In-Dels observed and 34.2% of the TEs (Additional file 1: Figure S3A; Additional file 2: Table S19). Comparisons of the density of variation between the RIVs and the core genome further confirmed higher genetic variation (2.91/10 kb *vs* 0.97/10 kb) and increased transposon sequences (2.99/10 kb *vs* 1.08/10 kb), but fewer genes (2.99/10 kb *vs* 1.93/10 kb) in the RIV sequences (Additional file 1: Figure S3B; Additional file 2: Table S19). Functional annotation revealed that the 861 genes encoded by the RIVs were involved in basic cellular processes such as cell division, protein binding, biosynthesis, and others (Additional file 1: Figure S4). Of the RIV genes, 79 (9.18%) were observed as under selection (i.e., with genetic variation), and the TFs (20%, 8 genes) displayed significant selection relative to other potential pathogenicity-related genes in RIVs (Additional file 2: Table S20). The TE-rich regions in the VdLs.16 genome therefore encode genes involved in regulatory processes and undergo mutations at rates higher than in regions with fewer TEs.

### Regions of increased variation are correlated with enrichment of the LTR/*Gypsy* TEs

**Fig. 5:**
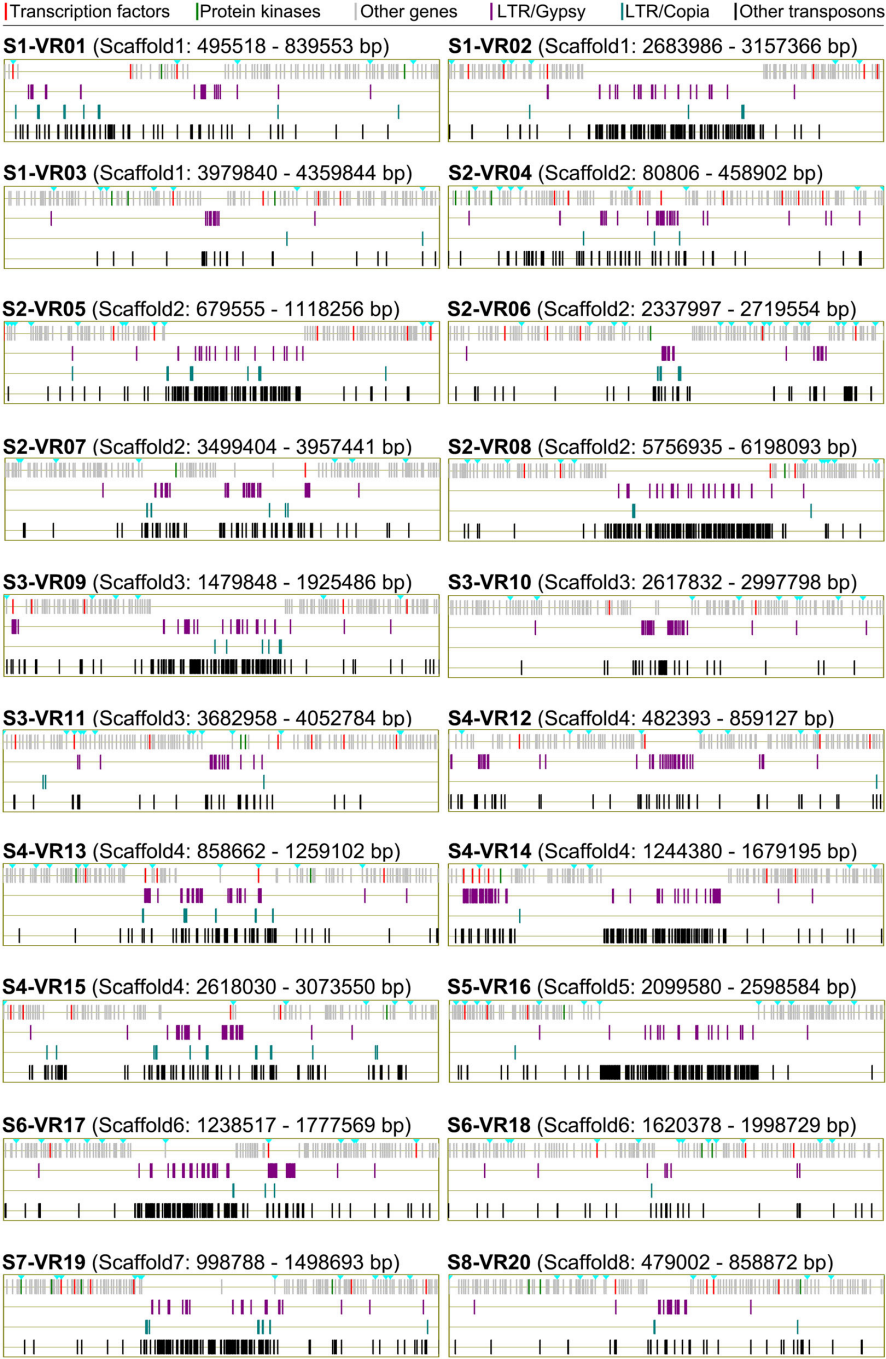
Annotation of the regions of increased genetic variation (RIVs) in *Verticillium dahliae*. The inverted triangle in aqua color represents the gene under selection in the RIVs designated as RIV01–RIV20

Analysis of the RIVs along with the 100-kb flanking sequences showed significantly enriched LTR/Gypsy type of TEs (Fig. 5). Because LTR/Gypsy elements are widespread in the RIVs, there may be fewer encoded genes in RIV, such as in S1-RIV02, S2-RIV08, S3-RIV09, and others (Fig. 5). In addition, even though the sequence length of RIVs comprised only 12.4% of the VdLs.16 genome, 41% (662 out of 1614) of the LTR/Gypsy transposons were located in the RIVs (Additional file 1: Figure S3A). Statistical analyses of the numbers of LTR/Gypsy revealed that the average number within the RIVs ranged between 1.88/10 kb and 2.94/10 kb, relative to 0.44/10 kb in the rest of VdLs.16 genome (Additional file 1: Figures S1 and S5A). Furthermore, the RIVs plus their 100-kb flanking sequences encode 2059 genes in total (861 genes encoded in RIVs) (Fig. 5; Additional file 2: Table S18). Of the 861 encoded in the RIVs, 187 genes were under selection in the VdLs.16 genome, and several RIVs were enriched with the genes under selection, including S1-RIV06, S2-RIV13, and S3-RIV18 (Fig. 5; Additional file 1: Figure S5B and S5C). These results suggest that RIVs are enriched with the LTR/Gypsy transposons, which may facilitate the enhancement of genetic variation in these particular regions of the *V. dahliae* genome.

### The SNPs and In-Dels fixed in RIVs are enriched in genes with signaling and gene regulatory functions

**Fig. 6:**
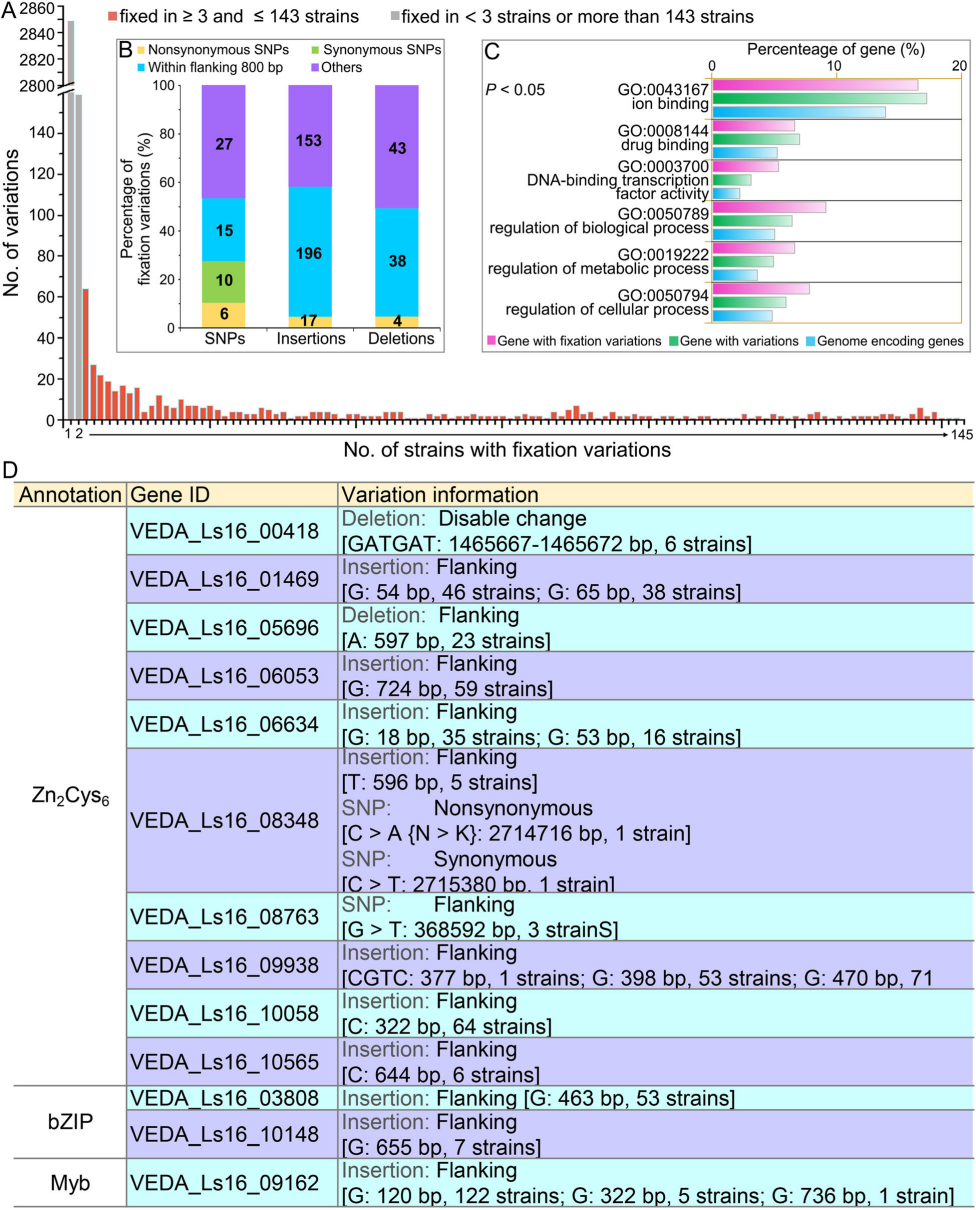
Analysis of the fixation of variation within a *Verticillium dahliae* population. **a** Statistics of the fixed genetic variations in the collection of the VdLs.16 population. The genetic variation that was representative of more than two strains and less than 144 strains (assumed sequence errors in the reference genome) defined fixed variations in the VdLs.16 population. **b** Classification of the fixed genetic variations. **c** Gene ontology (GO) annotation of the genes under selection with fixed genetic variations. The significant GO catalogs of genes under selection in the VdLs.16 population compared to the whole genome was determined by the Pearson chi-square test (*P* < 0.05). **d** Information on transcription factors with the fixed genetic variations

**Fig. 7:**
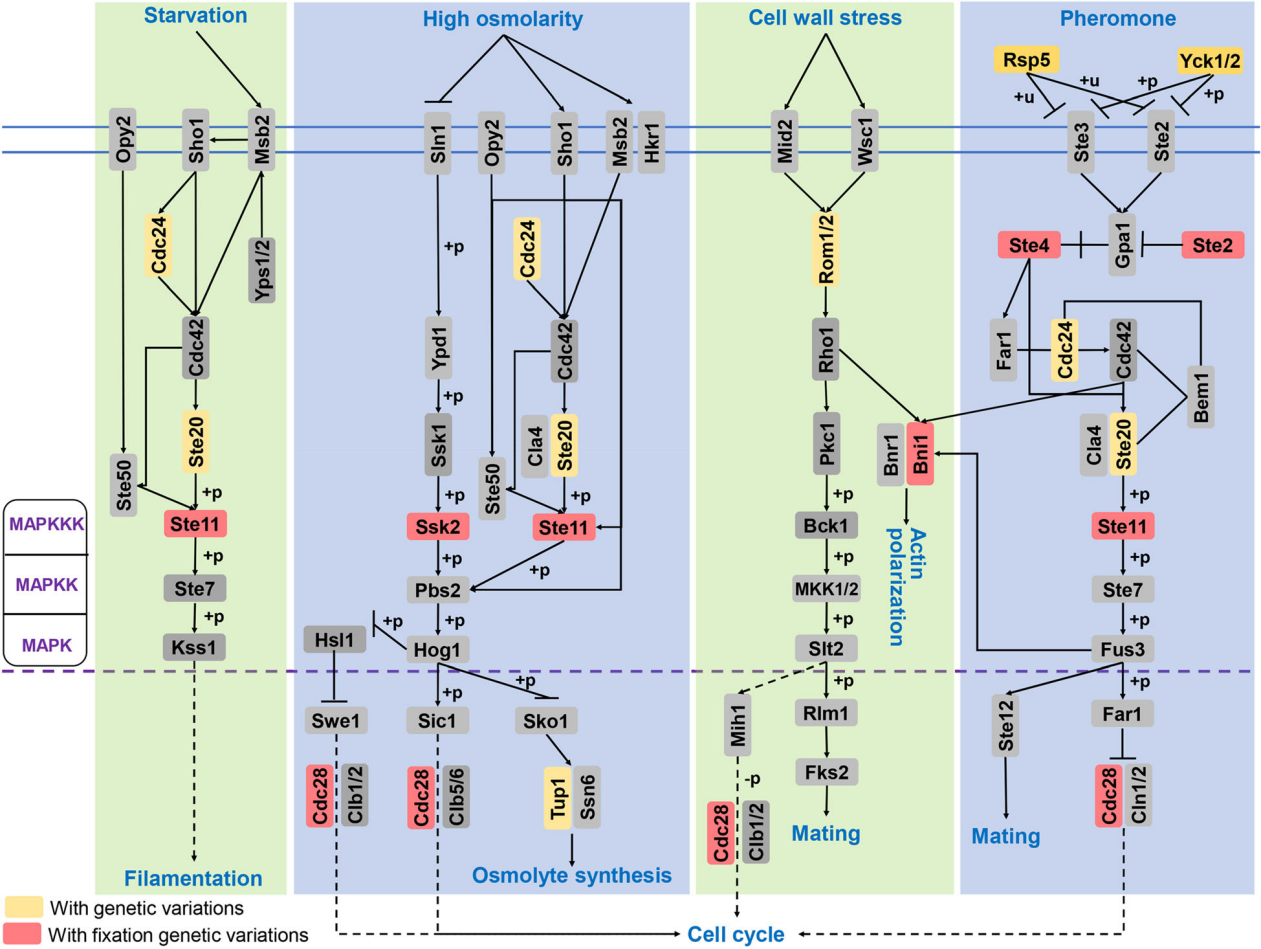
Network maps of genes under selection in mitogen-activated protein kinase (MAPK) signaling pathways. The model was drawn using genes under selection annotated using the KEGG database with the species *V. dahliae*. +p, phosphorylation; −p, dephosphorylation; +u, ubiquitylation

To examine genetic variation potentially caused by local adaptation in the field over time, the fixation of SNPs and In-Dels were assessed in the VdLs.16 population. The sequence divergence that co-occurred in more than two strains in addition to filtering variations co-occurring as sequencing errors were defined as fixed genetic variations. In total, excluding the genetic variation that occurs in solitary strains (85.48%, 3008 variations) and the variation co-present in more than 143 strains (two variations), 509 variants (including 58 SNPs, 366 insertions, and 85 deletions) were identified as fixed genetic variations in the VdLs.16 population (Fig. 6a; Additional file 1: Figure S6). Of these, 21 SNPs, 213 insertions, and 42 deletions were observed in the coding sequence or within 800 bp of flanking sequence (Fig. 6b), which yielded 243 genes under selection in the VdLs.16 population (Additional file 2: Table S17). Of these genes, 33 contained mutations in their coding sequences and 10 of these also possessed variations within the 800 bp flanking sequence (Additional file 1: Figure S1C). Gene ontology (GO) annotation revealed that genes under selection with fixed variations were significantly enriched in gene regulatory function mediated by TFs (*P* < 0.05), including DNA-binding transcription factor activity (GO:0003700, 13 genes), regulation of biological process (GO:0050789, 22 genes), and regulation of cellular process (GO:0050794, 19 genes) (Fig. 6c). Functional annotation showed that 13 TFs, including 10 Zn_2_Cys_6_, two bZIP, and one Myb transcription factor were under selection with fixed variations mainly in the 800 bp flanking sequences (Fig. 6d). Additionally, Kyoto Encyclopedia of Genes and Genomes (KEGG [46];) annotation of 243 genes with fixed variations revealed that 17 pathways were under selection for host adaptation, including starch and sucrose metabolism and mitogen-activated protein kinase (MAPK) signaling pathways that may be necessary for pathogenicity (Additional file 1: Figure S7; Additional file 2: Table S21). Of the genes with fixed variations, six encode PKs that are involved in MAPK signaling pathways, including Ste2, Ste4, Ste11, Ssk2, Bni1, and Cdc28 (Fig. 7). In addition, gene expression analysis of randomly selected genes encoding TFs and PKs (four genes for each) showed that all of them were significantly upregulated during infection of lettuce root (Additional file 1: Figure S8). Thus, the genes encoding regulatory and signaling functions, inferred by the TFs and PKs, accumulate significant variation under selection to gradually become fixed in *V. dahliae* over six seasons in the field.

## Discussion

With the advent of high-throughput sequencing, whole genomes under selection pressure and mechanisms of genome evolution have been studied both within and between closely related species under natural and managed ecosystems [47]. Such studies on isolates collected from highly variable natural habitats or from steady-state managed ecosystems have had difficulty in identifying genetic variations associated with local adaptations. We employed a closed managed agricultural ecosystem whereby *V. dahliae* race 1 strain was the only strain introduced into a field which was continuously cropped with lettuce for 6 years. The derivative race 1 population collected over a 6-year period allowed us to document the local adaptation of the parental VdLs.16 strain in a managed agricultural ecosystem. *Verticillium dahliae* accumulated genetic variations among the collected individuals, and these variations were associated with hypervariable transposon-rich regions encoding components of signal transduction and transcriptional regulation (Figs. 1 and 4), suggesting that incremental changes at the whole-genome level may continuously drive the pathogen adaptation over the longer term in the managed agricultural ecosystems. Though no such study can rule out the environmental factors on microbial adaptations in field populations, we know from anecdotal observations that the influence of environmental factors on *V. dahliae* evolution are seldom observed over the short term. More than 100 years of research on this pathogen has not produced evidence for environmental factors altering virulence or other biological characteristics. For instance, soil fumigation over a 50-year period in coastal California [48] has not generated novel pathogen virulence or variants with expanded host range or other altered biological characteristics. Thus, this is the first study to investigate the genetic variations in a soilborne pathogen such as *V. dahliae,* at the whole-genome level in the field, facilitating the understanding of how *V. dahliae* achieves local adaptation in a managed agricultural ecosystem. Because *V. dahliae* is a soilborne fungus that forms microsclerotia that survive up to 14 years in soil [27, 30], reproduces asexually [44, 49], and is thought to retain genetic stability over long periods, this study is particularly insightful.

Like other strictly asexual pathogens, *V. dahliae* lacks meiotic recombination associated with sexual lifestyles and has a reduced probability of fixing advantageous mutations [50]. In our study, the thousands of genetic variations in the derivative population of 145 isolates were discretely distributed, i.e., only a small proportion of the genetic variation (509 SNPs and In-Dels) were fixed in more than two isolates (Fig. 6a; Additional file 1: Figure S6). In addition, most of the genetic variations (36.9%) occurred in the gene-sparse regions enriched in transposon sequences (Figs. 4 and 5; Additional file 1: S3A; Additional file 2: Table S19), indicating that the generation of genetic variations likely are associated with TEs described in *V. dahliae* [18, 44]. In addition, scanning of the genetic variations in the VdLs.16 genome by step window analyses showed that the derivatives of VdLs.16 strains preserved at least one SNP or In-Del in almost all 100-kb-long windows (Fig. 4, except five windows), indicating that whole-genome selection had occurred in the race 1 strain in the managed agricultural system.

TEs are notoriously problematic with respect to genome assemblies and thus likely affect the accuracy of mutations detected in the derived population. To reduce biasing of the genetic variations caused by TEs, we performed whole-genome resequencing (more than 80-fold of genome size) that ensured high mapping depth (Additional file 2: Table S11) and collected only the most reliable variant bases (20 × depth). Most importantly, around 20% genetic variations were supported by the population that was fixed in at least three isolates but was unaltered in other isolates in the population (Fig. 6a; Additional file 1: Figure S1; Additional file 2: Table S16), which demonstrated that the genetic variations detected were reliable. In addition, we performed PCR to detect the accuracy of genetic variations that occurred in only one or two isolates, which confirmed that at least 75% of the genetic variations of random selections were indeed accurate (Additional file 1: Figure S9). Together, the genetic variations detected in the derived population were reliable.

The genetic variations were scattered across the whole genome but their occurrence as unfixed in the VdLs.16-derived population was unexpected (Fig. 4). As an asexual pathogen, *V. dahliae* proliferates clonally to maintain homozygosity [26]. Most genetic variations (58.48%) occurred in isolates collected during the first year, and new genetic variations were observed in subsequent years (Fig. 1a). Only small proportions of genetic variation (22 SNPs and 188 In-Dels) were repeatedly identified in the subsequent years (Fig. 1a). Strictly asexual pathogens such as *V. dahliae* may employ a “shotgun” approach to initiate genetic variations scattered over the whole genome to enhance the probability of fixing a limited number of mutations for adaptation in managed agricultural ecosystems. In this case, all of the genes that undergo mutation (Fig. 2) but are not fixed in the population (Fig. 6) are meaningful for understanding the selection and evolutionary tendencies of *V. dahliae* in managed agricultural ecosystems. However, the low ratio of stable genetic variation may be a result of genetic drift, similar to the dispersal rates and genetic drift observed in the potato late blight pathogen to adapt to the most abundant host genotype in an agricultural plant-pathosystem [51].

TEs with high mutagenic potential serve as an active site of evolution in several pathogens [16, 17] and lead to genome evolution in the agricultural ecosystems [14]. Similarly in *V. dahliae*, TEs likely act as major generators of *Verticillium* intra- and inter-specific genomic variation [18, 44], which are expected to play critical roles in genome adaptation in managed agricultural ecosystems. Although the genetic variations were scattered throughout the genome, the variations were significantly enriched in the transposon-rich and gene-sparse regions and were specifically associated with the LTR/Gypsy transposons (Figs. 4 and 5). The filamentous fungi have a unique tool, known as repeat-induced point mutation (RIP), to induce the genetic variations in repetitive sequences such as TEs [52], as some of the RIP protein machinery is conserved in *V. dahliae* [18] and *Neurospora crassa* [53]. Furthermore, RIP mutations affected members of the *Gypsy* but not the *Copia* superfamilies of retrotransposons in *V. dahliae* [18]. Therefore, the regions of increased variation probably correlate with the enrichment of TEs, especially with the LTR/*Gypsy* transposons. In addition, genetic variations within RIVs (as the “buffer region”) that consist of few coding genes and numerous transposons may also reduce the functional gene divergence and probably facilitate purges of mutations introduced by transposable elements. Therefore, our studies suggested that as an asexual pathogen, *V. dahliae* also significantly accumulates genetic variations in an agricultural ecosystem, and these are linked with transposable elements.

Deciphering the local adaptations in agricultural systems is extremely complicated given the range of factors, including temperature, humidity, pH, and host plant, against which selection needs to occur. Local adaptation in other pathogen populations demonstrate that virulence may be governed by quantitative trait loci but not avirulence genes/secreted proteins, resulting in many abiotic factors that contribute to the outcome of the field/host adaptation [3], including proteins involved in in planta signaling, regulation of pathogen gene expression, invasive growth, and the formation of specific infection structures [5]. Intriguingly, the candidate genes showing high selection pressure in race 1 *V. dahliae* populations challenged in the managed agricultural ecosystem were significantly enriched in the regulatory factors, especially the transcription factors and protein kinases (Figs. 3 and 6). In the *V. dahliae* lettuce strain VdLs.17, certain families of transcription factors and TEs are enriched in lineage-specific (LS) regions in the genome relative to the core genomic sequence [24], and these LS regions are more variable and thought to confer adaptability [24, 44]. Though the LS regions described previously are distinct from the RIVs identified in this study, they share commonality in that TEs were enriched in both, contributing to their variability. Transcription factors also displayed significant family expansion and contraction within *V. dahliae*, with greater numbers in VdLs.16 (Additional file 2: Table S9). Moreover, multiple transcription factors contribute to virulence in *V. dahliae* [54–57]. Therefore, genes encoding regulatory proteins involved in signaling and transcriptional regulation may be important for local adaptation of *V. dahliae* in a managed agricultural ecosystem.

The *V. dahliae* race 1 population in our managed agricultural ecosystem in this study was continuously challenged with the race 1-resistant lettuce during the test period, which indicated that the pathogen probably underwent divergence owing to host selection. In our race 1 population from a managed agricultural ecosystem, 987 genes (including 604 genes with genetic variations in the potential 800 bp gene promoter) were defined as genes under selection pressure (Fig. 2), which is also caused by the selection from host resistance. Generally, the coevolution that occurs in plant-pathogen interactions is thought to be governed largely by the highly specific interactions between avirulence and resistance gene products, in which case, pathogens acquire virulence or pathogenicity on a resistant host genotype primarily by mutations in the matching avirulence genes [58]. For instance, the *Blumeria graminis* avirulence gene *AVR*_*a*_ includes SNPs that resulted in a gain of virulence on barley [59]. Such cases are common in pathogens under selection for locally adapted traits in the agricultural ecosystems, especially when pathogen populations are under strong directional selection for local adaptation to fungicides [60]. However, the avirulence factor *Ave1* or any of the predicted genes encoding secreted proteins were not altered under selection pressure when challenged with lettuce carrying the *Vr1* locus for resistance (Figs. 3b and 4; Additional file 1: Figure S2; Additional file 2: Table S12). This may appear to be in conflict with the knowledge that secreted proteins are under high selection pressure during host-pathogen interactions. During the coevolution of host-pathogen interactions, the host is the strongest driver of evolution in a pathogen, and hence, infection-related genes are under strong selection pressure in pathogen genomes [5]. Secreted proteins from pathogens, including avirulence factors/effectors, are key players in interactions with host plants, often enduring greater positive selective pressure than other genes [12, 58, 61]. Such is the case for the selection of small secreted proteins in *Z. tritici* in response to avirulent interactions on wheat cultivars carrying *Stb6* for *Z. tritici* resistance [62]. Previous studies [63] also showed that the dynamic virulence-related regions of *V. dahliae* display enhanced sequence conservation relative to other regions. However, an avirulence gene selected to contribute to the fitness of the pathogen on a susceptible host genotype can be an important driver of local adaptation [3], which probably is the reason why pathogen populations do not employ “arms race” evolution as a priority to facilitate extensive prevalence of the avirulent phenotype. Similar studies also found that the evolutionary trajectory is largely determined by spatially heterogeneous biotic and abiotic selection pressures, and not necessarily a member of the gene-for-gene interactions as in *Rhynchosporium commune* [1], *Zymoseptoria tritici* [11], and *Parastagonospora nodorum* [12]. As determined in this study, the upstream genes involved in signaling and transcriptional regulation but not those encoding secreted proteins endured strong selection for local adaptation in *V. dahliae* in a managed agricultural ecosystem.

## Conclusions

In conclusion, our study showed that the strictly asexual pathogen *V. dahliae* evolves at a whole genome scale to accumulate genetic variations (SNPs and In-Dels) nonrandomly in hypervariable regions associated with transposon enrichment in the managed agricultural ecosystems. The increased variation of genes encoding products obviously involved in signaling and transcriptional regulation may play important roles in local adaptation in *V. dahliae* in managed agricultural ecosystems.

## Methods

### Disease nursery establishment

All isolates used in this study were obtained from a Verticillium wilt disease nursery at the USDA Research Station in Salinas, California. The disease nursery was established with the *V. dahliae* race 1 isolate, VdLs.16 [36]. Briefly, the field with no previous history of *V. dahliae* infestation was fumigated using methyl bromide (67%) and chloropicrin (33%) mixture at 361 kg/ha in 2009 before nursery establishment. Following fumigation, the absence of residual *V. dahliae* inoculum in the fumigated field was confirmed from soil assays [64]. Lettuce seedlings of susceptible cultivar Salinas (*vr1*) were first grown in a greenhouse in germination trays filled with sterilized potting mix in the spring and fall seasons of 2009 and 2010. The 14-day-old seedlings were inoculated in trays with 3 mL of 1 × 10^7^ conidia/mL suspension of isolate VdLs.16 (collected from a commercial lettuce field in Watsonville, CA in 1996) per plug and repeated twice within a week. Following the last inoculation, seedlings were grown for two additional weeks in the greenhouse and moved outside the greenhouse for acclimatization for 10 days before transplanting. Inoculated seedlings were transplanted into the field at a spacing of approximately 28 cm within and between two seed lines on each bed. Plants were grown to maturity and incorporated into the soil by disking. This process of planting inoculated lettuce seedlings, growing the crop to maturity, and incorporating the residue elevated the soil inoculum levels to > 100 microsclerotia g^−1^ soil. This high inoculum density was maintained throughout the study years 2010–2015 by planting susceptible lines each season interspersed with the plants with race 1 resistance.

Between 2010 and 2015, an early and intermediate generation of families and inbred lines derived from a cross between La Brillante (race 1 resistant, *Vr1*) and other cultivars of the iceberg, romaine, and leaf type lettuce were screened in approximately 45% of the test plots in the nursery. Plant introductions with resistance to race 1 or partial resistance to race 2 isolates in a greenhouse were planted in another 5% of test plots. The remainder of the plots were occupied by susceptible germplasm. Planting patterns were completely randomized each year.

### Derivative isolate collections

Following disease evaluations, roots from arbitrarily selected diseased plants were collected in spring 2010, 2012, 2014, and 2015. The symptomatic root samples representative of the entire disease nursery were processed for fungal isolation by plating surface-sterilized (with 10% NaOCl for 3 min followed by three washings with sterile distilled water) root tissue on semi-selective NP-10 medium [64] enriched with streptomycin sulfate and chlortetracycline HCl at 100 ppm. *V. dahliae*-like colonies that emerged from the plated tissue were transferred to fresh plates containing NP-10 and purified as described in previous study [36]. The pure cultures were further processed either for long-term storage in 25% glycerol at − 80 °C or for DNA extraction.

### Isolate cultivation and DNA preparation

From the cultures of actively growing isolates on PDA, two to three 3-mm-diameter plugs were transferred into 50 mL potato dextrose broth (PDB) and grown for 1 week on laboratory benches at room temperature (23 ± 2 °C). The fungal mycelium was harvested after 1 week, washed, lyophilized, and processed for total genomic DNA extraction using FastDNA Kit (MP Biomedical, Santa Ana, CA, USA). Total DNA was quantified, diluted, and used for downstream processing. The high-quality genomic DNA was extracted using the Quick-DNA^TM^ Fugnal/Bacterial Midiprep Kit (Zymo Research, Orange, CA, USA) according to the manufacturer’s protocol.

### Genome sequencing and assembly

Isolate VdLs.16 collected from a commercial lettuce field in Watsonville, CA, in 1996, was used for de novo genome sequencing. The library construction, sequencing, reads filtering, and assembly procedure of the VdLs.16 reference genome was performed as described in our previous study [65]. For resequencing of the derivative VdLs.16 population, a total of ~ 3 Gb paired-end short reads (PE = 150 bp) were generated by the Illumina Hiseq 2000 platform.

### Gene prediction and annotation

Protein-coding genes in the VdLs.16 genome, and the gapless reference genome of VdLs.17 (GenBank assembly accession: GCA_000952015.1) and JR2 (GCA_000400815.2) [40], were predicted using a combination of de novo-based and homology-based approaches as described previously [21]. The secreted proteins of sequenced genomes were predicted from WoLF PSORT [66], SignalP4.1 [67], TMHMM 2.0 [68] and Phobius [69] as described in Klosterman et al. [43]. The Small cysteine-rich protein (SCRPs) were obtained by using a custom Perl script that counted proteins with < 400 amino acids and ≥ 4 cysteine residues. The annotation of putative CAZymes was performed using the HMM-based routine of the Carbohydrate-Active-EnZymes database [70]. CAZymes involved in plant cell wall degradation were collated according to the classification methods described previously [71, 72]. The homologs of known pathogen-host interaction (PHI) factors were predicted using the PHI-base (Version 4.7, http://www.phi-base.org) [73]. The PKs were predicted by running HMM searches locally with Kinomer (Version 1.0) [74]. The transcription factors were designated by the conserved domains that were predicted through the InterPro database.

### Characterization of transposons

Transposable elements were identified by RepeatMasker (open 3.2.8, detailed parameters: -no_is, -norna, -engine, -s, -parallel = 1, used Repbase version 15.08) and RepeatProteinMask (-noLowSimple, -pvalue = 1e-4) (http://www.repeatmasker.org). The output files were summarized using a custom *Perl* script.

### SNP and In-Del calling

To identify SNPs within the sequenced isolates, Illumina clean reads for VdLs.16 population were aligned to the VdLs.16 reference assembly using the BWA program (-o 1 -e 63 -i 90 -L -k 2 -l 31 -t 4 -q 10) [75]. SNPs were identified and filtered using the following parameters of GATK (GenomeAnalysisTK-3.4-0; --filterExpression "DP < 20 || QUAL<30.0 || QD < 2.0 || MQ < 40.0 || FS > 60.0 || SOR > 3.0" --filterName "Filter") [76]; the length of the variant base is less than or equal to 10 bp; the depth of the variant base is more than 20.

To identify In-Dels within the sequenced isolates, Illumina clean reads for the VdLs.16 population were aligned to the VdLs.16 reference assembly using a BWA program (-o 1 -e 63 -i 90 -L -k 2 -l 31 -t 4 -q 10) [75]. In-Dels were identified and filtered using the following parameters of GATK (GenomeAnalysisTK-3.4-0; --filterExpression "DP < 20 || QUAL<30.0 || QD < 2.0 || FS > 200.0 || SOR > 10.0" --filterName "Filter"). The length of the variant base is less than or equal to 10 bp; the depth of the variant base is more than 20.

The sequence divergence of *Ave1* in the VdLs.16-derived population was determined by mapping the reads of the VdLs.16 progeny population to the *Ave1* gene with 1-kb flanking sequence, and the depth of each base was calculated and standardized by the base with highest depth (set as 100%).

### PCR detection of the genetic variations

To determine the accuracy of genetic variations in the resequenced whole-genome data, 24 genetic variations in individual strains were randomly selected for PCR amplification using primers designed to produce a 400–600-bp amplicon (Additional file 2: Table S22). The PCR program consisted of an initial denaturation step at 94 °C for 10 min, followed by 35 cycles at 94 °C for 30 s, 54 °C for 45 s, and 72 °C for 30 s with the primers using the corresponding sample. All the amplicons were sequenced by classical method. The accuracy of genetic variations were detected by sequence alignment of the sequenced PCR amplicon with the refine genome sequence.

### RT-qPCR detection of genes related to the genetic variations in the VdLs16 population

Sterile lettuce seedlings were grown in tissue culture dishes with roseite as the substrate in liquid Murashige and Skoog medium at 25 °C in a greenhouse under an alternating 16-h light and 8-h dark regime. VdLs.16 strain was cultured on potato dextrose agar (PDA) medium and then was transferred to liquid Cazepk medium and incubated for 4 days at 25 °C. A 50 μL conidial (5 × 10^6^ conidia/mL) suspension was spread on the PDA medium and cultured for three additional days. By 1 week, the plates with lettuce seedlings were covered by *V. dahliae* growth and served as the induced strain. The induced strains were collected at 36 h and 60 h, respectively. The uninduced VdLs.16 strain was used as the control. The AxyPreP Multisource Total RNA Miniprep Kit (Axygen, USA) was used to isolate total RNA, and first-strand cDNA was synthesized using a TransScript II One-Step gDNA Removal and cDNA Synthesis SuperMix Kit according to the manufacturer’s instructions (TransGen, China). Reverse transcription-quantitative PCR (RT-qPCR) was performed under the following conditions: an initial 95 °C denaturation step for 5 min, followed by denaturation for 30 s at 95 °C, annealing for 30 s at 60 °C and extension for 30 s at 72 °C for 40 cycles. The *V. dahliae* elongation factor 1α (*EF-1α*) was used as the endogenous reference. Relative transcripts levels of genes were determined using the 2^−∆∆CT^ method [77]. Unpaired Student’s *t* tests were performed to determine statistical significance at *P* < 0.01 to make pairwise comparison of treatments.

## Supplementary Information

**Additional file 1: Supplementary Figure S1.** Genetic variations identified within the genome, gene coding sequences, and gene flanking sequences of strain VdLs.16 of *Verticillium dahliae*. (A) SNPs, In-Dels, and fixed variations in the genomic sequences of the VdLs.16 population. (B) Numbers of genes with genetic variations in their coding sequences and gene flanking sequences. (C) Venn diagram characterization of the numbers of genes with fixed genetic variations in gene coding and flanking sequences. **Supplementary Figure S2.** Identification of the genetic variation at the *Ave1* locus of *Verticillium dahliae*. The reads of the VdLs.16 progeny population were mapped to the *Ave1* gene with 1-kb flanking sequence, and the depth of each base was calculated and standardized by the base with highest depth (set as 100). The red square box represents the region of the *Ave1* gene. **Supplementary Figure S3.** Population genomics comparison between the regions showing enriched variation in the VdLs.16 genome. (A) Genetic variations in regions compared to the VdLs.16 population. (B) The density of genetic variations and genome annotation contents in the regions of the VdLs.16 genome with increased genetic variation (RIVs). The density of variations was calculated by the average in 10 kb windows by the total number of variations in the full genome sequence. **Supplementary Figure S4.** Genes encoded in the regions of increased genetic variation within the VdLs.16 genome. The significant GO catalogs of genes encoded in the regions of increased genetic variation were selected by the Pearson *Chi-Square* test (*P* < 0.05). **Supplementary Figure S5.** Distribution of LTR/Gypsy transposons and gene enrichment in the regions of increased genetic variations in strain VdLs.16 of *Verticillium dahliae*. (A) Enrichment of the LTR/Gypsy transposons in the regions of increased variation. The enrichment of the LTR/Gypsy transposons was assessed by the number of LTR/Gypsy transposons per sequence length of 10 kb. The density of coding genes and genes under selection was assessed (B) within the regions and (C), within the regions plus 100-kb flanking sequence. **Supplementary Figure S6.** Distribution of the fixed genetic variations in the VdLs.16 population of *Verticillium dahliae*. The orange color represents the strains with the genetic variations compared with the VdLs.16 reference genome. **Supplementary Figure S7.** Annotation of the genes from strain VdLs.16 of *Verticillium dahliae* under selection in starch and sucrose metabolic pathways. The pink box represents the genes under selection mapped to the pathway by KEGG database annotation. **Supplementary Figure S8.** Expression levels of genes with fixed genetic variations under induction by lettuce. (A) and (B) The PDA plates with one-week-old lettuce seedlings were inoculated with a 50 μL conidial (5 × 10^6^ conidia/mL) from the VdLs.16 strain. The strain was harvested at 36 h and 60 h after co-cultivation with lettuce seedlings. RT-qPCR was performed to determine the expression levels of randomly selected genes (encoding transcription factors and protein kinases) with fixed genetic variations, relative to *V. dahliae* elongation factor 1-α (*EF-1α*). Error bars represent standard errors and asterisks represent statistical significance at *P* < 0.01 (**) and *P* < 0.001 (***), respectively, based on unpaired Student’s *t*-tests. **Supplementary Figure S9.** PCR detection of the accuracy of genetic variations determined by resequencing. (A) 24 genetic variations determined in individual strain were selected randomly for PCR detection. (B) Determined the genetic variation by sequence alignment of sequencing of PCR amplicon with the refine genome sequence. Red triangle represents the genetic variation (C) Gel of PCR products amplified from 24 individual samples.

**Additional file 2: Supplementary Table S1.** Clean data of VdLs.16 genome sequenced by PacBio RS II and Illumina technologies. **Supplementary Table S2.** Prediction of the transposons in the sequenced genomes. **Supplementary Table S3.** The CAZymes in VdLs.16 genome. **Supplementary Table S4.** The CAZymes sub-families in VdLs.16 genome. **Supplementary Table S5.** The CAZymes in VdLs.16 genome. **Supplementary Table S6.** The secreted CAZymes sub-families in VdLs.16 genome. **Supplementary Table S7.** The protein kinase subfamilies VdLs.16 genome. **Supplementary Table S8.** The PHI homologs in VdLs.16 genome. **Supplementary Table S9.** The transcription factors in the VdLs.16 genome. **Supplementary Table S10.** Information of VdLs16 population collected from disease nursey. **Supplementary Table S11.** Sequencing data of the VdLs.16 population. **Supplementary Table S12.** Coverage and depth of the resequencing VdLs.16 population. **Supplementary Table S13.** Determination of the genotype of VdLs.16 population by gene markers. **Supplementary Table S14.** Statistics of the SNP variations in VdLs.16 population. **Supplementary Table S15.** Statistics of the insertion-deletion variations in VdLs.16 population. **Supplementary Table S16.** Identification of the genetic variations in VdLs.16 population. **Supplementary Table S17.** Functional annotation of the genetic variations in VdLs.16 population. **Supplementary Table S18.** Information of the genetic variation regions under selective in VdLs.16 genome. **Supplementary Table S19.** Statistics of the genetic variations density between genome and genetic variation regions. **Supplementary Table S20.** Statistics of genes under selection in the regions of increased genetic variation. **Supplementary Table S21.** Pathway annotation by KEGG database with *Verticillium dahliae* model. **Supplementary Table S22.** Primers used in this study.

## Data Availability

This Whole Genome Shotgun project of VdLs.16 and the version used in this study has been deposited at DDBJ/ENA/GenBank under the accession JABBIU000000000 (https://www.ncbi.nlm.nih.gov/nuccore/JABBIU000000000.1/) [78]. The sequencing data of VdLs.16 strains collected from the lettuce field among 2010 to 2015 used in this study has been deposited at DDBJ/ENA/GenBank under the accession PRJNA625638 (https://dataview.ncbi.nlm.nih.gov/object/PRJNA625638) [79].
