# Supplementary material for: Dynamics of Verticillium dahliae race 1 population under managed agricultural ecosystems
Source: BMC Biol. 2021 Jun 25;19:131. doi: 10.1186/s12915-021-01061-w (PMC8235872; doi:10.1186/s12915-021-01061-w)
Supplement: Supplementary file 1 — Additional file 1: Supplementary Figure S1. Genetic variations identified within the genome, gene coding sequences, and gene flanking sequences of strain VdLs.16 of Verticillium dahliae. (A) SNPs, In-Dels, and fixed variations in the genomic sequences of the VdLs.16 population. (B) Numbers of genes with genetic variations in their coding sequences and gene flanking sequences. (C) Venn diagram characterization of the numbers of genes with fixed genetic variations in gene coding and flanking sequences. Supplementary Figure S2. Identification of the genetic variation at the Ave1 locus of Verticillium dahliae. The reads of the VdLs.16 progeny population were mapped to the Ave1 gene with 1-kb flanking sequence, and the depth of each base was calculated and standardized by the base with highest depth (set as 100). The red square box represents the region of the Ave1 gene. Supplementary Figure S3. Population genomics comparison between the regions showing enriched variation in the VdLs.16 genome. (A) Genetic variations in regions compared to the VdLs.16 population. (B) The density of genetic variations and genome annotation contents in the regions of the VdLs.16 genome with increased genetic variation (RIVs). The density of variations was calculated by the average in 10 kb windows by the total number of variations in the full genome sequence. Supplementary Figure S4. Genes encoded in the regions of increased genetic variation within the VdLs.16 genome. The significant GO catalogs of genes encoded in the regions of increased genetic variation were selected by the Pearson Chi-Square test (P < 0.05). Supplementary Figure S5. Distribution of LTR/Gypsy transposons and gene enrichment in the regions of increased genetic variations in strain VdLs.16 of Verticillium dahliae. (A) Enrichment of the LTR/Gypsy transposons in the regions of increased variation. The enrichment of the LTR/Gypsy transposons was assessed by the number of LTR/Gypsy transposons per sequence length of 10 kb. [file 12915_2021_1061_MOESM1_ESM.docx]

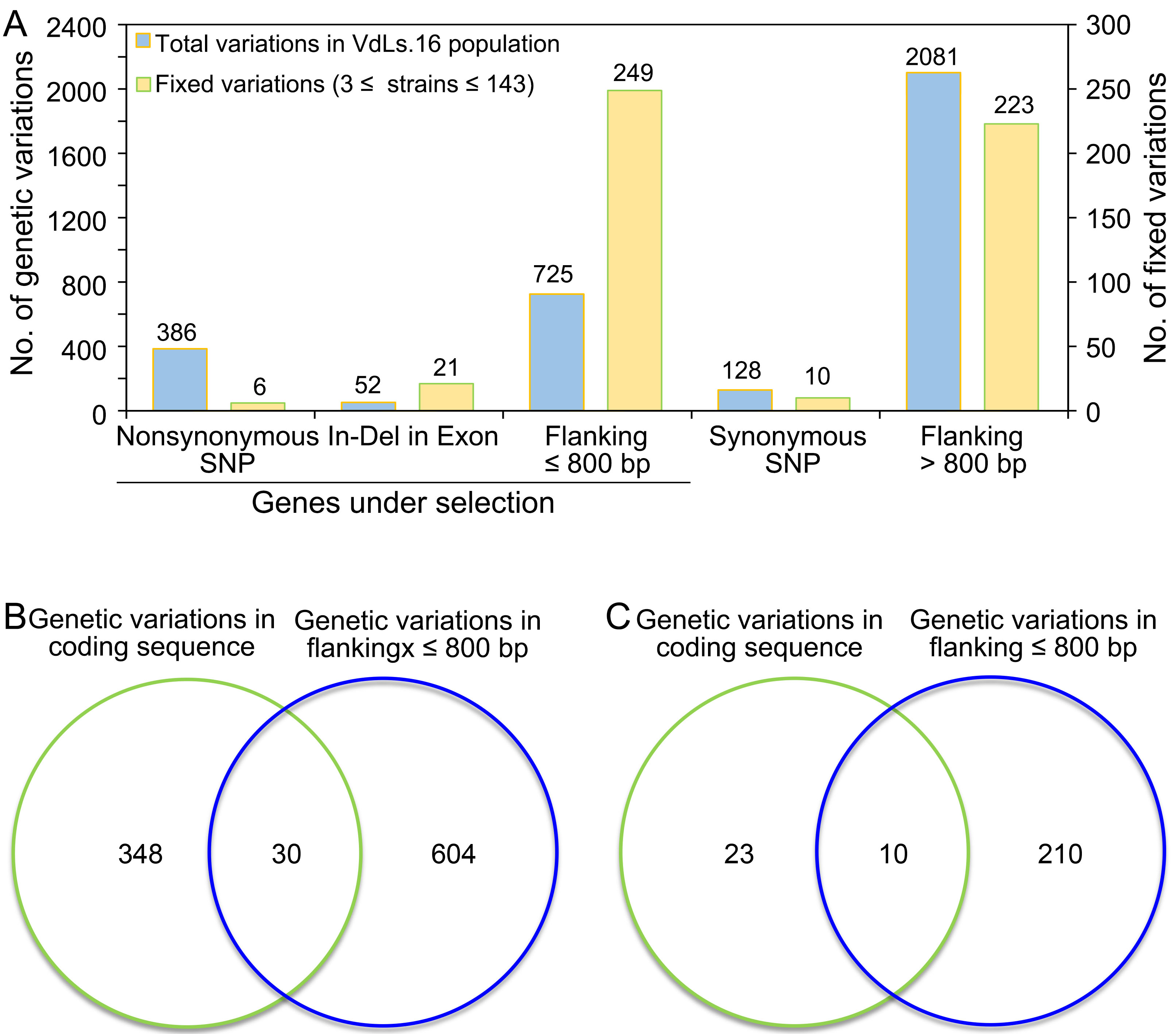


**Supplementary Fig. S1. Genetic variations identified within the genome, gene coding sequences, and gene flanking sequences of strain VdLs.16 of *Verticillium dahliae*.** **(A)** SNPs, In-Dels, and fixed variations in the genomic sequences of the VdLs.16 population. **(B)** Numbers of genes with genetic variations in their coding sequences and gene flanking sequences. **(C)** Venn diagram characterization of the numbers of genes with fixed genetic variations in coding sequences and gene flanking sequences.


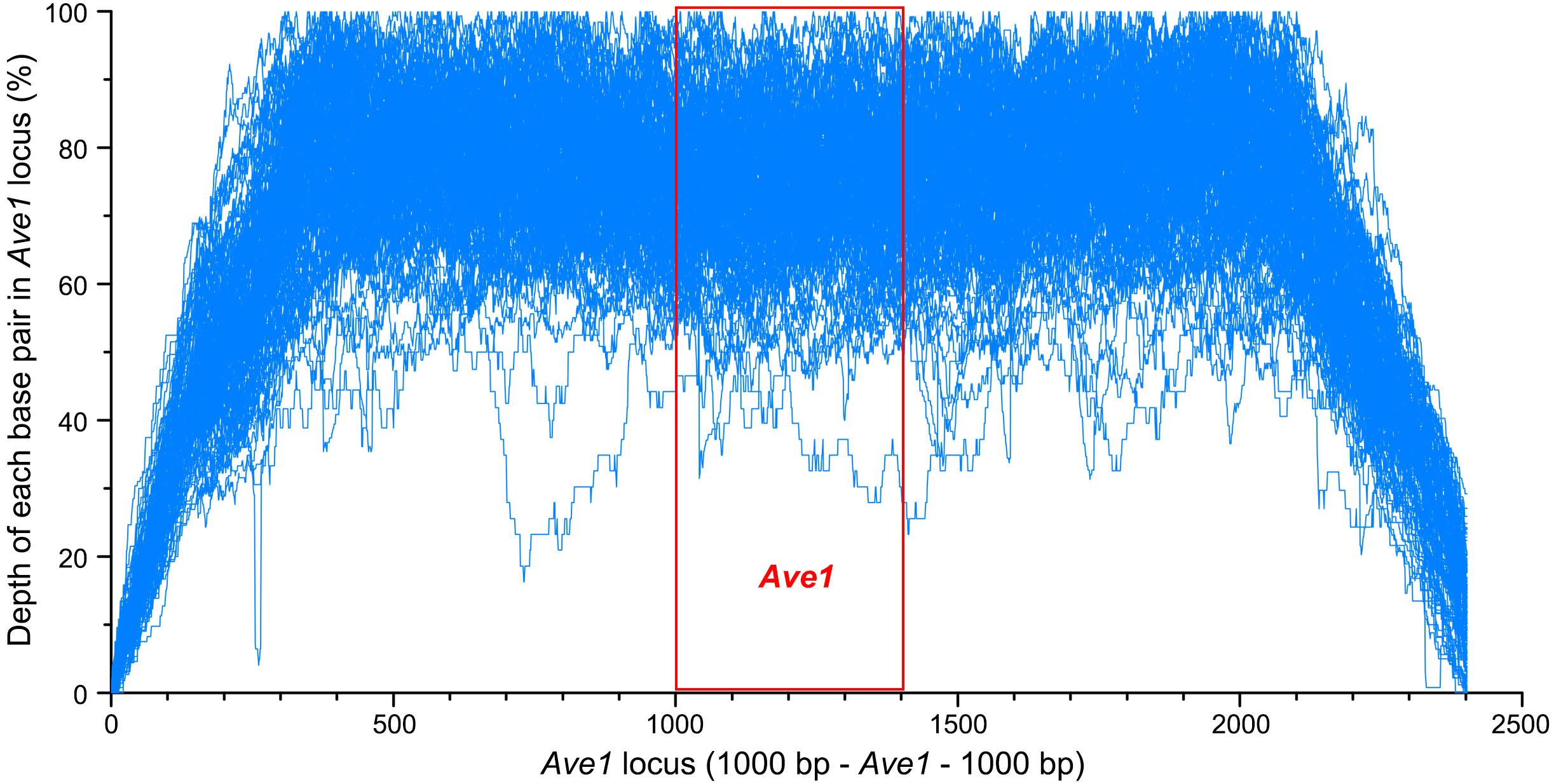


**Supplementary Fig. S2. Identification of the genetic variation at the *Ave1* locus of *Verticillium dahliae*.** The reads of VdLs.16 progeny population was mapped to the *Ave1* gene with 1 kb flanking sequence, and the depth of each base was caculated and standardized by the base with highest depth (set as 100). The red square box represent the region of *Ave1* gene.


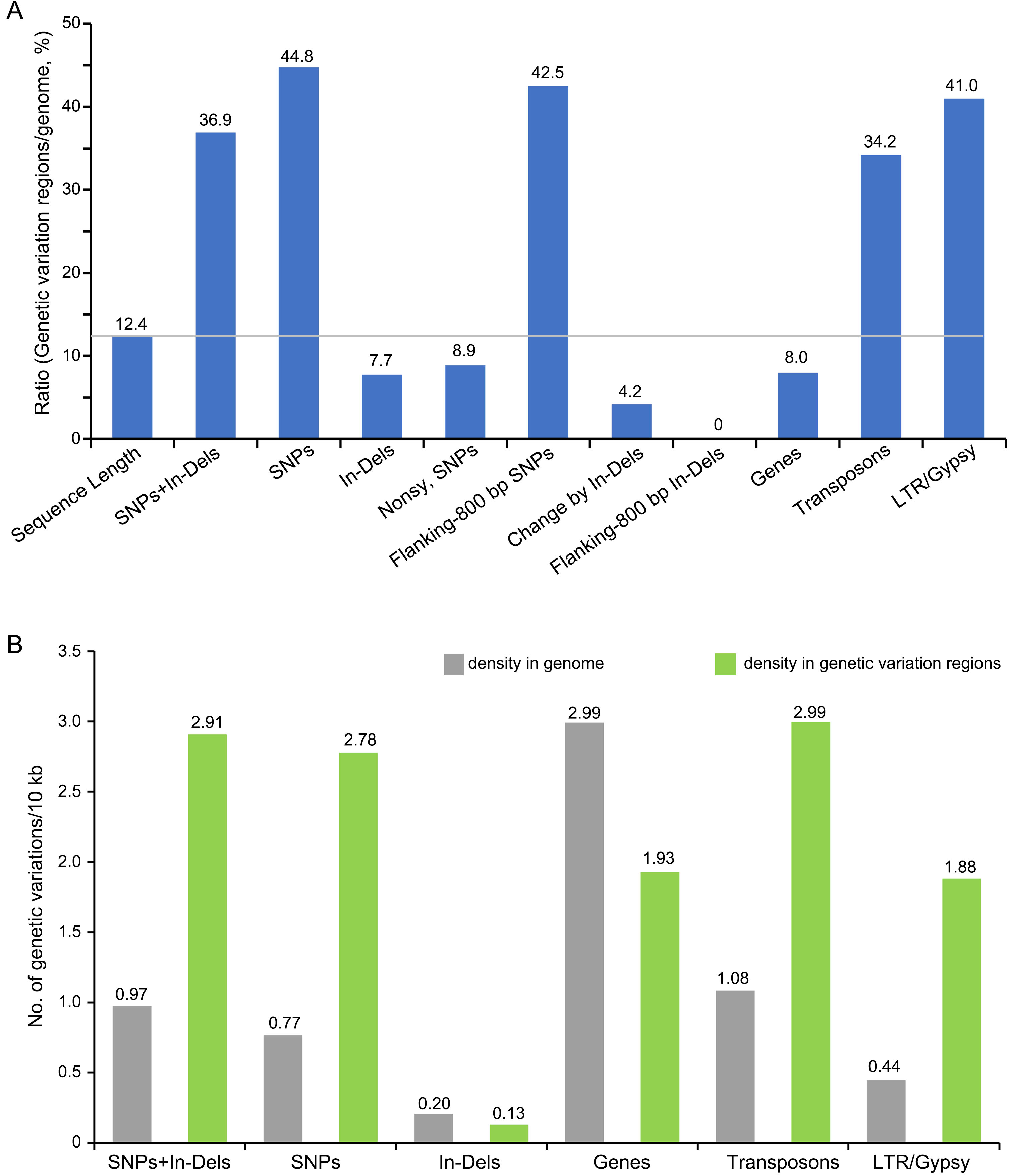


**Supplementary Fig. S3. Population genomics comparison between the regions showing enriched variation in the VdLs.16 genome.** **(A)** Genetic variations in regions compared to the VdLs.16 population. **(B)** The density of genetic variations and genome annotation contents in the regions of the VdLs.16 genome with increased genetic variation (RIVs). The density was calculated by the average converted to 10 kb by the total number of variations in the full sequence.


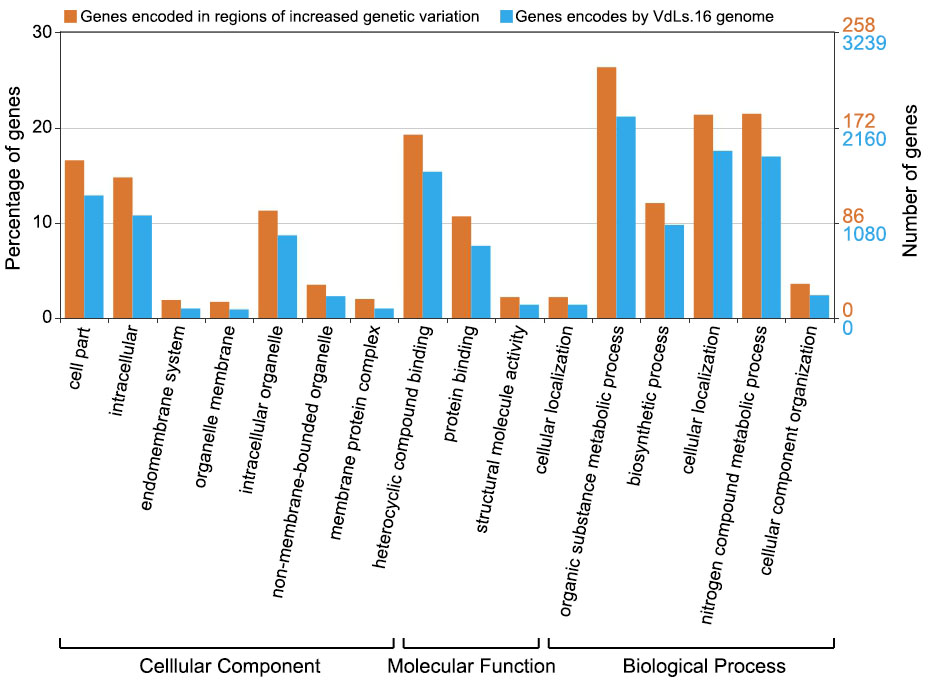


**Supplementary Fig. S4. Genes encoded in the regions of increased genetic variation within the VdLs.16 genome.** The significant GO catalogs of genes encoded in the regions of increased genetic variation was selected by the Pearson *Chi-Square* test (*P* < 0.05).


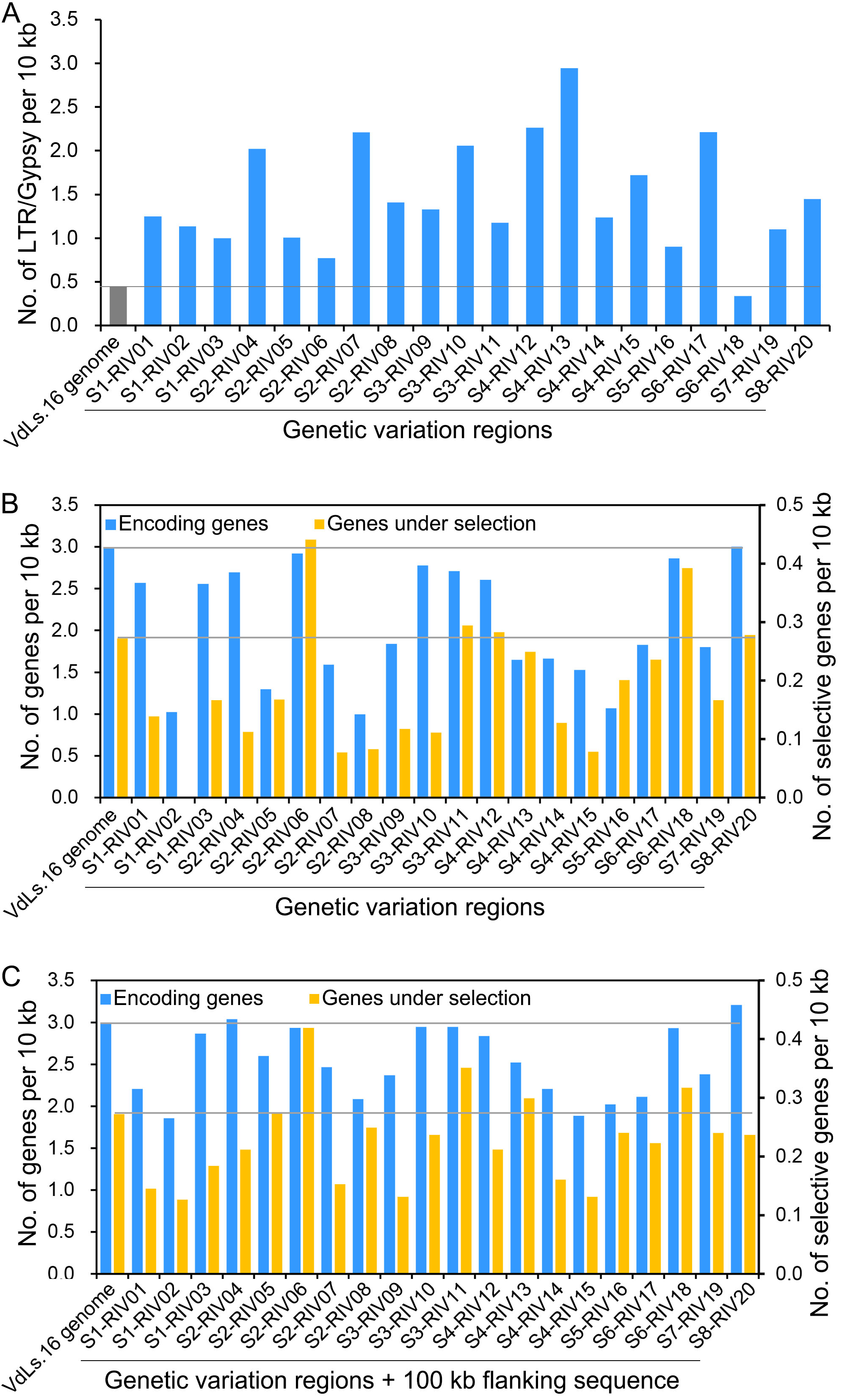


**Supplementary Fig. S5. Distribution of LTR/Gyspy transposons and gene enrichment in the regions of increased genetic variations in strain VdLs.16 of *Verticillium dahliae*. (A)** Enrichment of the LTR/Gypsy transposons in the regions of increased variation. The enrichment of the LTR/Gyspy transposons was assessed by the number of LTR/Gyspy transposons per sequence length of 10 kb. The density of coding genes and genes under selection was assessed **(B)** within the regions and **(C)**, within the regions plus100 kb flanking sequence.


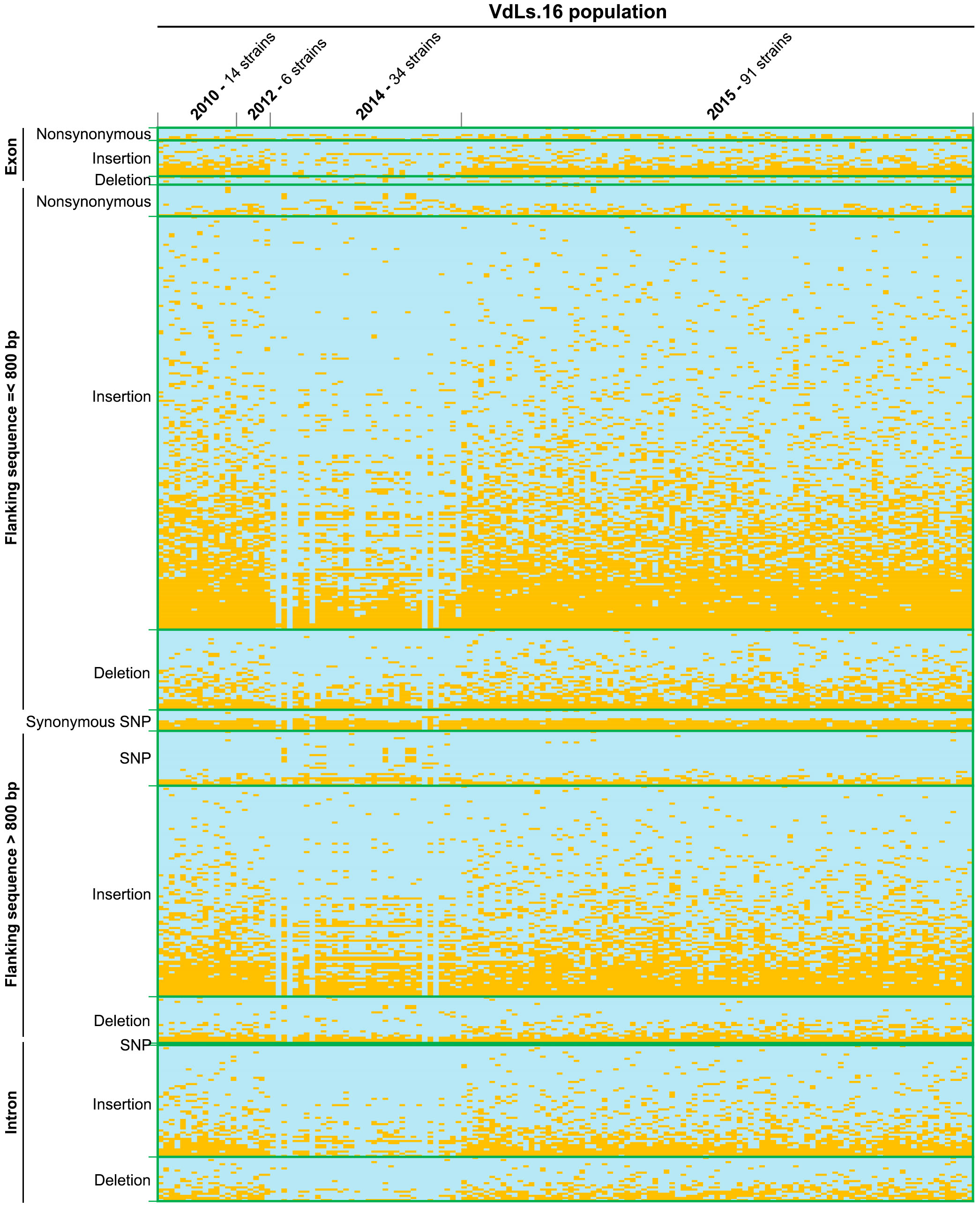


**Supplementary Fig. S6. Distribution of the fixed genetic variations in the VdLs.16 population of *Verticillium dahliae*.** The orange color represents the strains with the genetic variations compared with the VdLs.16 reference genome.


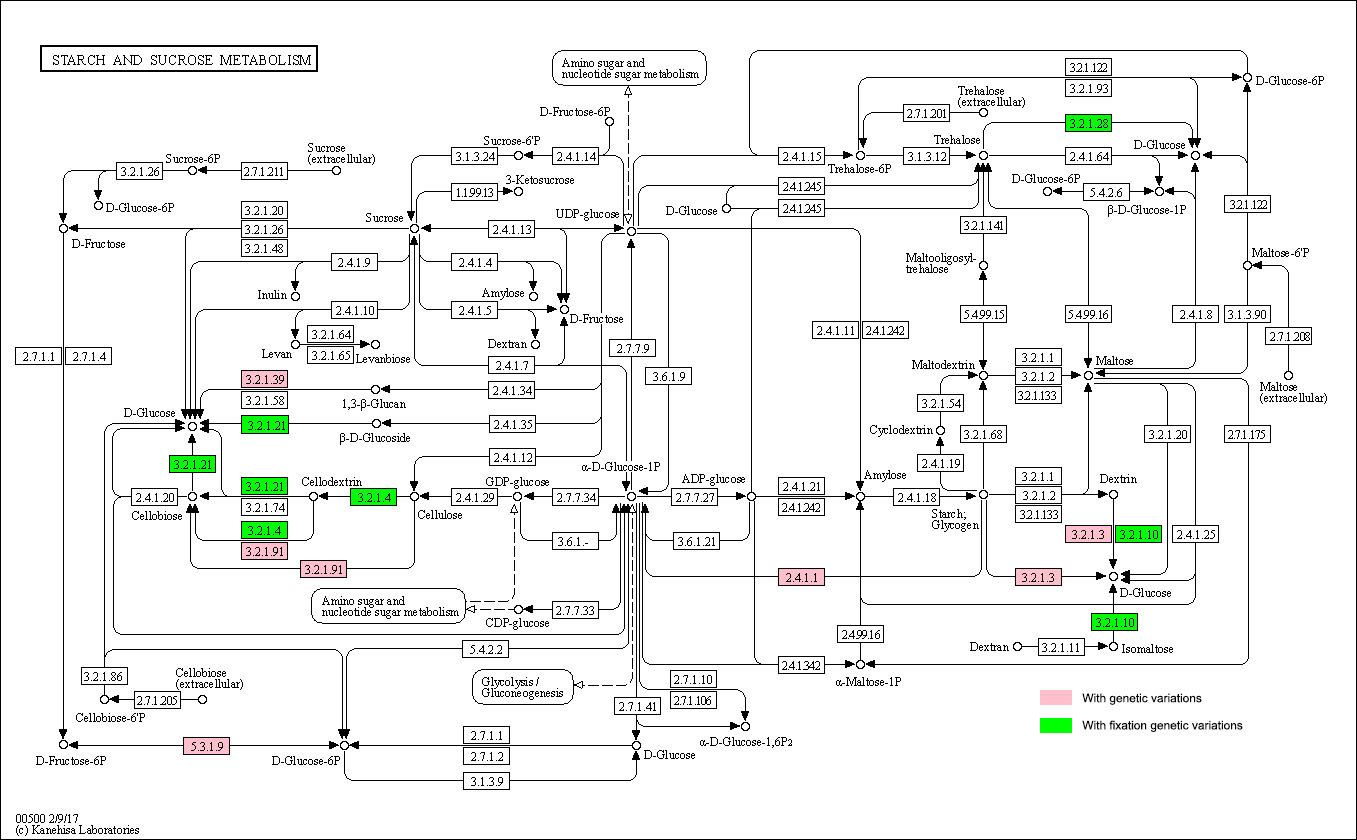


**Supplementary Fig. S7. Annotation of the genes from strain VdLs.16 of *Verticillium dahliae* under selection in starch and sucrose metabolic pathways.** The pink box represents the genes under selection mapped to the pathway by KEGG database annotation.

**
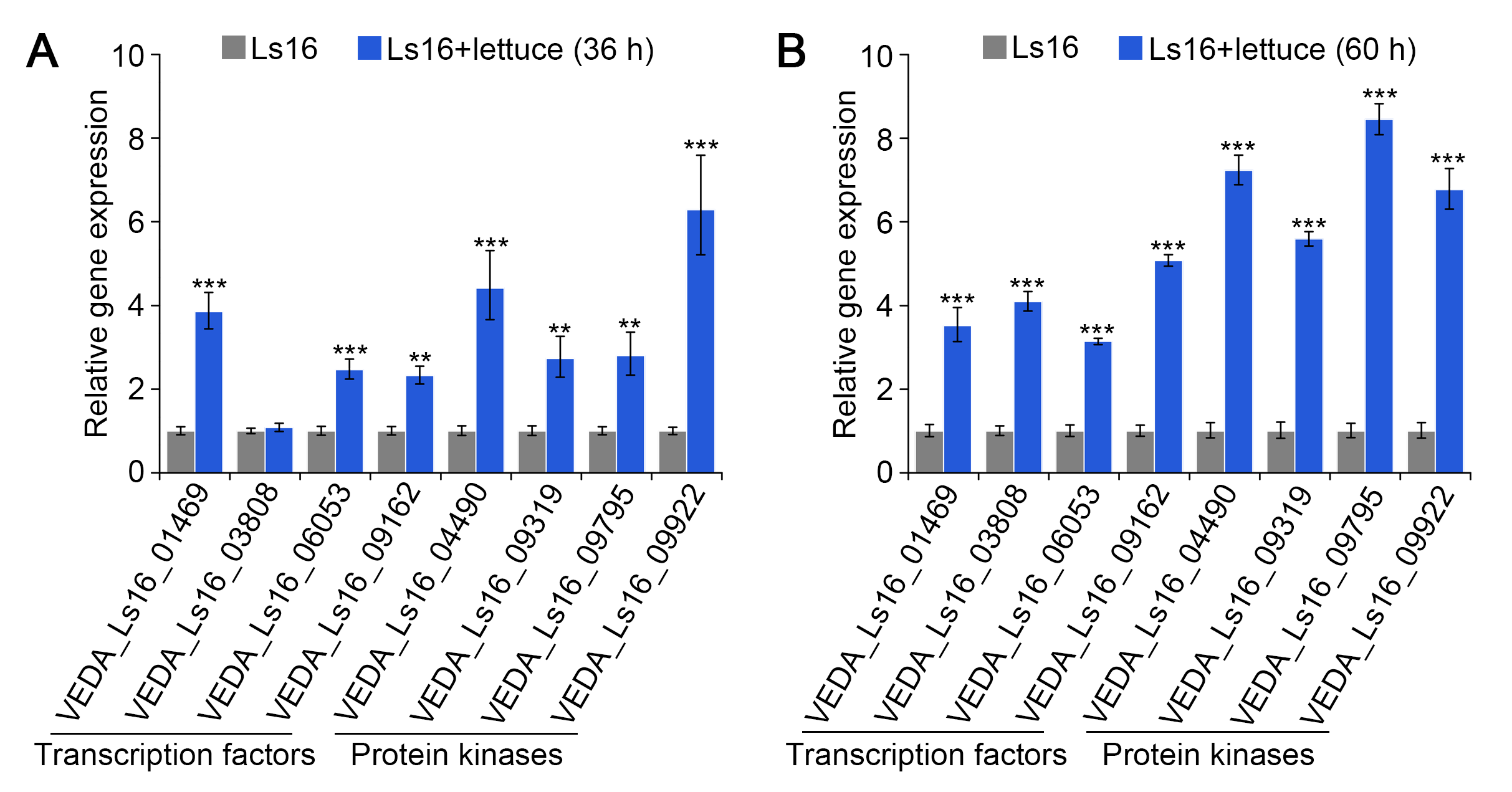
**

**Supplementary Fig. S8. Detected the expression level of genes with fixed genetic variations under induction by the host plants of lettuce.** (**A**) and (**B**)

The pre-culturing of VdLs.16 strains on PDA plates were induced by covering with one-week-old lettuce seedlings, and the strain was harvested at 36 h and 60 h after induction, respectively. RT-qPCR was performed to determine the expression levels of random selected genes (encodes transcription factors and protein kinases) with fixed genetic variations, involved relative to *V. dahliae* elongation factor 1-α (*EF-1α*). Error bars represent standard errors and asterisks represent statistical significance at *P* < 0.01 (**) and *P* < 0.001 (***), respectively, based on unpaired Student’s *t*-tests.


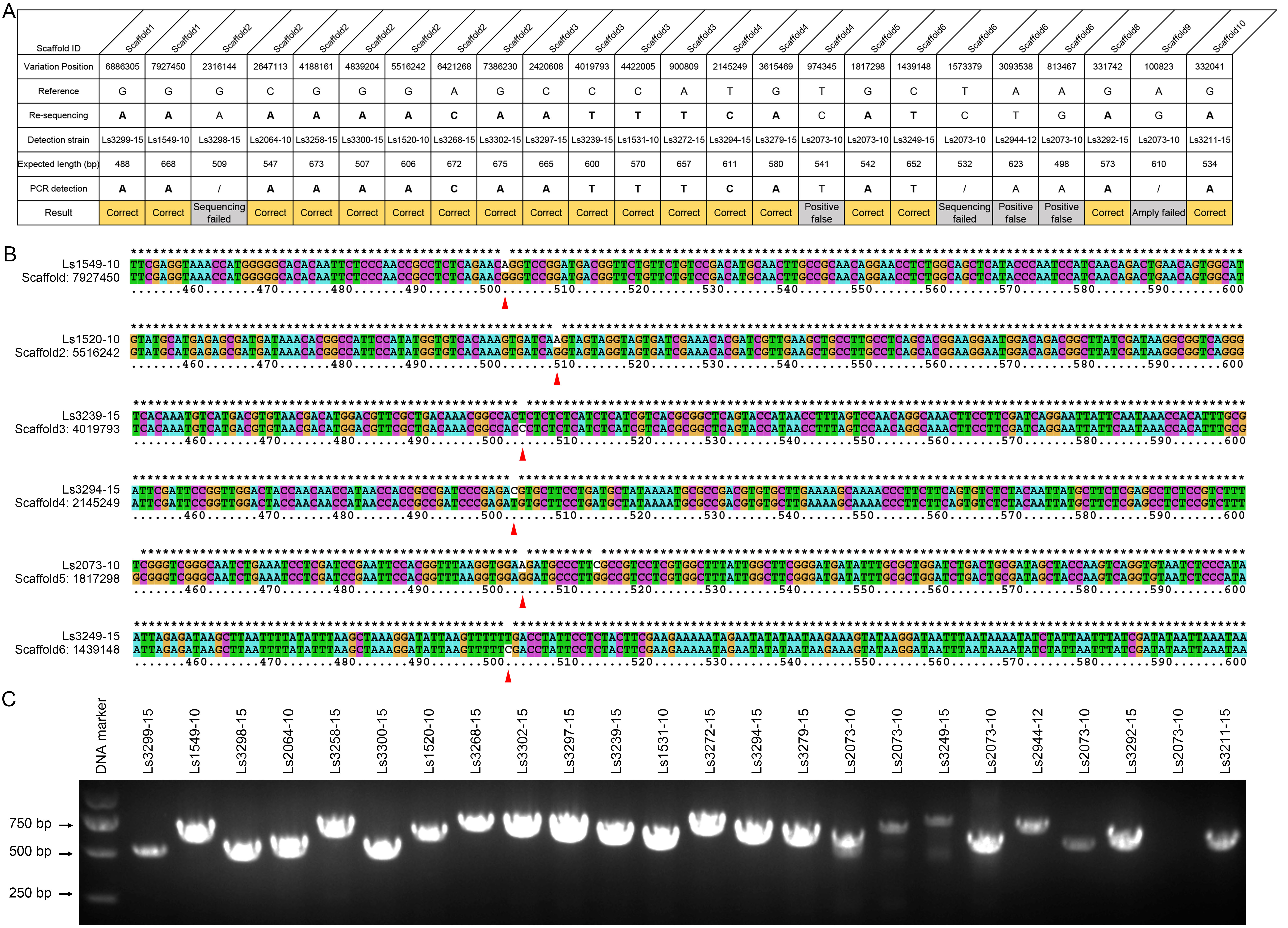


**Supplementary Fig. S9. PCR detection of the accuracy of genetic variations determined by re-sequencing.** (**A**) 24 genetic variations determined in individual strain was selected in randomly for PCR detection. (**B**) Determined the genetic variation by sequence aligement of sequencing of PCR amplicon with the refence genome sequence. Red triangle represents the genetic variation (**C**) Gel of PCR production from 24 individual samples.
